# Design, Synthesis, and Antimicrobial Activity Evaluation of Ciprofloxacin—Indole Hybrids

**DOI:** 10.3390/molecules28176325

**Published:** 2023-08-29

**Authors:** Mingxia Song, Yi Hua, Yuxin Liu, Xunli Xiao, Haihong Yu, Xianqing Deng

**Affiliations:** 1 Affiliated Hospital of Jinggangshan University, Ji’an 343000, China; mxsong@jgsu.edu.cn (M.S.);; 2 Health Science Center, Jinggangshan University, Ji’an 343009, China; 3 Center for Clinical Medicine Research of Jinggangshan University, Jinggangshan University, Ji’an 343009, China

**Keywords:** antibacterial, AMR, ciprofloxacin, indole, hybrid

## Abstract

With the overuse and misuse of antimicrobial drugs, antibacterial resistance is becoming a critical global health problem. New antibacterial agents are effective measures for overcoming the crisis of drug resistance. In this paper, a novel set of ciprofloxacin-indole/acetophenone hybrids was designed, synthesized, and structurally elucidated with the help of NMR and high-resolution mass spectrometry. The in vitro antibacterial activities of these hybrids against gram-positive and gram-negative pathogens, including four multidrug-resistant clinical isolates, were evaluated and compared with those of the parent drug ciprofloxacin (CIP). All the target compounds (MIC = 0.0625–32 μg/mL) exhibited excellent inhibitory activity against the strains tested. Among them, **3a** (MIC = 0.25–8 μg/mL) showed comparable or slightly less potent activity than CIP. The most active hybrid, **8b** (MIC = 0.0626–1 μg/mL), showed equal or higher activity than CIP. Moreover, compound **8b** showed superior bactericidal capability to CIP, with undetectably low resistance frequencies. Furthermore, molecular docking studies conducted showed that **8b** and CIP had a similar binding mode to DNA gyrase (*Staphylocouccus aureus*). Thus, hybrids **3a** and **8b** could act as a platform for further investigations.

## 1. Introduction

It is the law of nature that bacteria develop drug resistance to antibiotics. In recent decades, bacteria have developed drug resistance rapidly due to antibiotic overuse and misuse in animals, humans, and the environment, which has become a critical global health problem [1]. In 2020, antimicrobial resistance (AMR) has been listed among the top ten concerns in the field of public health by the World Health Organization [2]. AMR is a threat to humans, animals, plants, and the environment and causes significant disease burden [3,4]. A WHO report released in 2023 highlighted progress, but also remaining gaps, in ensuring a robust pipeline of antibiotic treatments to combat AMR [5]. Faced with the situation of increasing AMR and limited available antibiotics, there is an urgent need to develop potent and novel antibacterial drug candidates.

Ciprofloxacin (CIP), as one of the second-generation fluoroquinolones with a broad spectrum of activity, was patented in 1980 and approved in 1987. It possesses excellent pharmacokinetic properties and few side effects, and thus is used to treat multifarious bacterial infections. The World Health Organization classifies it as critically important for human medicine [6]. In 2019, it was the 113th-most-commonly prescribed medication in the United States, with more than 5 million prescriptions [7]. However, like other antimicrobial drugs, bacterial resistance to CIP develops quickly, making it increasingly ineffective. To enhance the antibiotic property of fluoroquinolones and relieve the problem of bacterial drug resistance, a large number of CIP derivatives have been prepared as potential antibacterial drug candidates in recent decades [8,9]. The majority of them are combined on the piperazine moiety [10,11]. Most of these derivatives showed weak or equivalent antibacterial activity to CIP. The battle to find new antibacterial agents against drug-resistant bacteria is endless.

Indole is a multifunctional active skeleton widely used in the field of drug research, which can bind to many kinds of receptors and enzymes and exhibit various biological activities, such as anticancer [12,13], anticonvulsant [14,15], antifungal [16,17], antitubercular [18,19], and antibacterial activities [20,21,22]. Currently, hybrids containing indole were reported frequently for their antimicrobial activity against a train of clinical pathogenic strains including drug-resistant strains, which demonstrated the potential of indole as a useful moiety for developing new antibacterial agents [21,22,23,24,25]. Indole structural scaffolds could act on DNA gyrase like CIP does, so hybridization of indole with CIP has the potential to enhance the DNA gyrase inhibition activity and strengthen antibacterial activity as a consequence [26].

**Figure 1:**
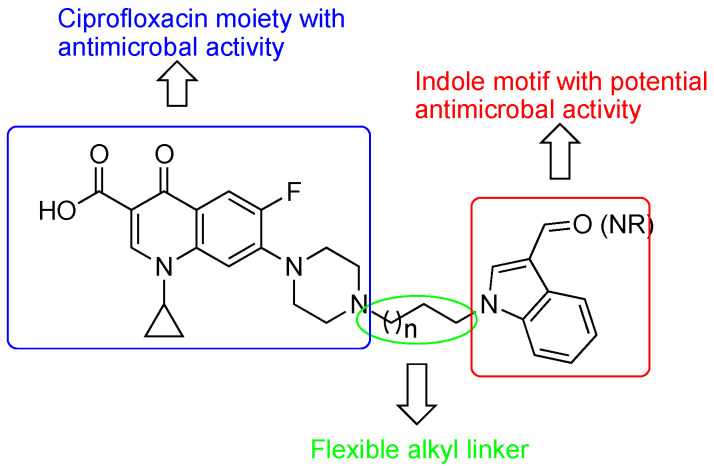
Design strategy for ciprofloxacin (CIP)–indole hybrids.

In view of the above-mentioned facts, a novel series of alkyl-tethered cipro–indole hybrids—**2**, **3a–d**, **5a–b**, and **6a–b**—was designed, prepared, characterized, and investigated for their antibacterial activities against representative clinical pathogenic bacteria (Figure 1). In the designed skeleton, flexible propylene was first selected to ensure the binding validity of the two active fragments [27]. To obtain more effective antibacterial compounds and enrich the existing structure–activity relationship, the indole group was altered into acetophenone. The corresponding CIP derivatives **7a–b** and **8a–b** were prepared and investigated for their antibacterial activity.

## 2. Results and Discussion

### 2.1. Chemistry

**Scheme 1:**
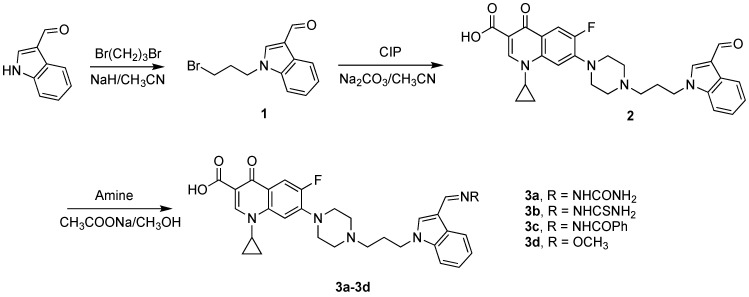
The synthetic route to prepare compounds **3a–3d**.

**Scheme 2:**
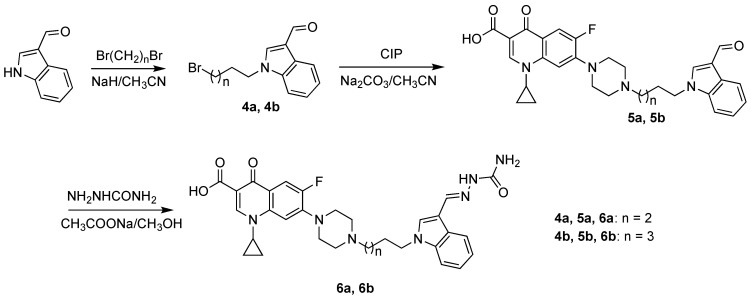
The synthetic route to prepare compounds **5a**, **5b**, **6a**, and **6b**.

**Scheme 3:**
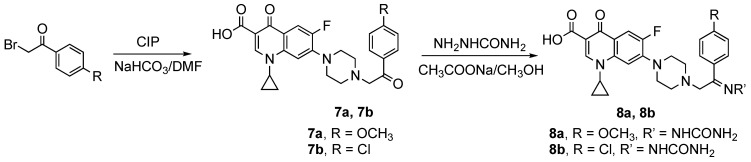
The synthetic route to prepare compounds **7a**, **7b**, **8a**, and **8b**.

The synthetic process of the CIP-contained hybrids was depicted in Scheme 1, Scheme 2 and Scheme 3. Indole-3-carboxaldehyde was treated with 1,3-dibromopropane in acetonitrile in the presence of sodium hydride to give intermediate **1**, which was reacted with CIP (98%, Macklin Inc., Shanghai, China) in the presence of Na_2_CO_3_ to give compound **2**. Compound **2** reacted with semicarbazide, thiosemicarbazide, benzoyl hydrazine, and O-methylhydroxylamine to give compounds **3a**, **3b**, **3c**, and **3d**, respectively. Compounds **4a** and **4b** were prepared using the same method as compound **1** by replacing the 1,3-dibromopropane with 1,4-dibromobutane, and 1,5-dibromopentane respectively. Correspondingly, compounds **5a–5b**, and **6a–6b** were prepared using the same method as compounds **2**, and **3**, respectively. Compounds **7a** and **7b** were prepared via the reaction of bromophenone and CIP in DMF in the presence of NaHCO_3_. Compounds **7a** and **7b** were reacted with semicarbazide in methanol in the presence of CH_3_CO_2_Na to obtain compounds **8a** and **8b**, respectively. The target compounds were identified via NMR and MS spectrometry (See Supplementary Materials for details).

### 2.2. Pharmacology

#### 2.2.1. Antibacterial Activity

The antibacterial activity of the desired CIP-contained hybrids against clinically important pathogens including clinical isolates of multidrug-resistant strains was investigated. The minimum concentration of compounds required to produce 90% inhibition of bacterial growth was defined as the minimum inhibitory concentration (MIC) and is reported in Table 1 and Table 2.

As shown in Table 1, CIP–indole **2** displayed potent antibacterial properties against the tested gram-positive and gram-negative strains, with MICs ranging from 0.5 to 8 μg/mL. However, the antibacterial activities of this compound were lower than the parent drug, CIP. To enhance the activity, the antibacterial active fragments semicarbazide, thiosemicarbazide, benzoyl hydrazine, and methoxyamine were assembled to compound **2** to obtain compounds **3a–3d**. Compounds **3a–3d** displayed potent antibacterial potency against the tested bacteria, with MIC ranging from 0.25 to 32 μg/mL. Among them, compound **3a** coupled with semicarbazide moiety was the most promising one, with MIC of 0.25–4 μg/mL against all tested strains, which was slightly less active than or comparable to the parent drug, CIP.

In order to explore the action of link length on the antibacterial property of CIP–indole hybrids, two semicarbazide derivatives (**6a**, **6b**) with different lengths of linkers were prepared. The antibacterial activity was investigated and compared for the semicarbazide-contained CIP–indole hybrids **6a**, **6b** and their synthetic intermediates **5a**, **5b**. It seems that hybrids with butyl (**5a** and **6a**) are more active than hybrids with pentyl (**5b** and **6b**), and the propyl was the optimum length between the CIP and indole moiety in this study.

To obtain more effective antibacterial compounds and enrich the existing structure–activity relationship, CIP–acetophenone hybrids containing the active fragment semicarbazide were prepared. The hybrids **8a**, **8b** and their synthetic intermediates **7a**, **7b** were investigated for their antibacterial activity. The four hybrids displayed excellent antibacterial activities with MIC ranging from 0.625 to 8 μg/mL. Compound **8b** emerged as the most active hybrid, showing the highest antibacterial activity, especially against clinical pathogens *S. aureus* CMCC 25923 with MIC of 0.0625 μg/mL, which was four-fold more potent than the parent drug CIP (MIC: 0.25 μg/mL). The MIC values of this compound against other strains were relatively equivalent to that of CIP and were lower than norfloxacin and penicillin.

We conducted additional assessments on the inhibitory potential of the CIP hybrids (**2**, **3a–d**, **5a–b**, **6a–b**, **7a–b**, **8a–b**) against various clinical isolates of multidrug-resistant (MDR) bacterial strains. As can be seen in Table 2, all synthesized hybrids presented good antibacterial potency against the above strains, with MICs ranging from 0.25 to 32 μg/mL. All compounds are less active than the parent drug, CIP, with the exception of **8b**, but more active than penicillin. CIP–acetophenone hybrid **8b** with semicarbazide and Cl substituents demonstrated the highest level of inhibitory potency against MDR strains. The MIC values of this compound were equivalent to that of CIP and were lower than norfloxacin and penicillin.

#### 2.2.2. Propensity for the Development of Bacterial Resistance

**Figure 2:**
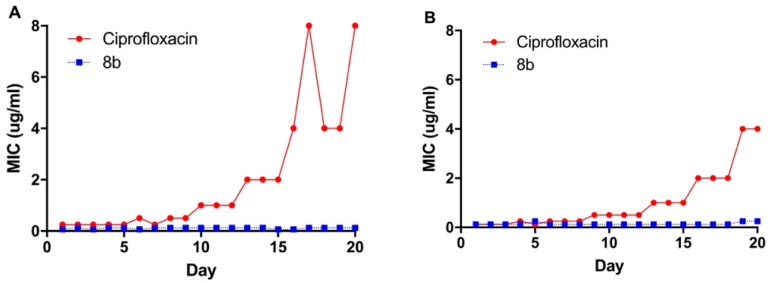
Propensity of the development of bacterial resistance toward compound **8b** and CIP ((**A**) for *S. aureus*; (**B**) for *E. coli*).

The resistance of bacteria to antibiotics is a major issue in today’s era [28,29,30]. Therefore, it is necessary to investigate the potential emergence of bacterial resistance towards the antibacterial candidates. To assess the propensity for bacterial resistance development, we evaluated the representative compound **8b** against *S. aureus* and *E. coli*, with CIP used as a reference drug. Compound **8b** and CIP were repeatedly exposed to bacteria at their sub-MIC values to allow for resistance development. Resistance was defined as a greater than four-fold increase in the original MIC [31]. As shown in Figure 2, no significant change in the MIC value of compound **8b** was noticed over 20 generations for *S. aureus* or *E. coli*. In contrast, the MIC of CIP increased approximately 32-fold and 16-fold for CIP over the same 20 generations. These findings indicate that compound **8b** has no propensity for the development of bacterial resistance within the experimental strains and time period.

#### 2.2.3. Evaluation of Bacterial Resistance Development

**Figure 3:**
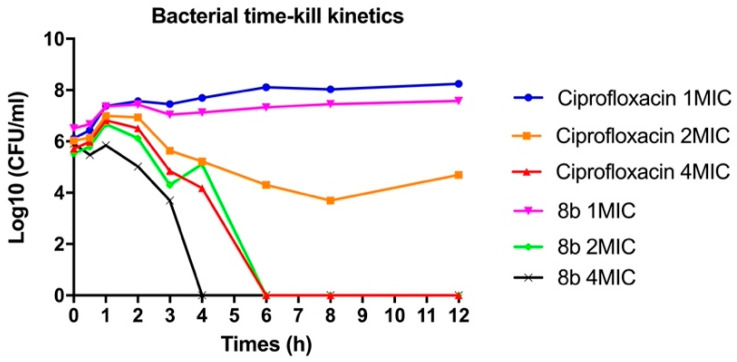
Bactericidal activities of compound **8b** and ciprofloxacin against MRSA.

In order to investigate the bactericidal activity of these hybrids, an in vitro time-kill assay was conducted using the representative compound **8b** against methicillin-resistant *S. aureus* (MRSA). Bactericidal activity was evaluated at three concentrations of **8b** (1 MIC, 2 MIC, and 4 MIC). CIP was employed as a positive control for comparison purposes in the in vitro time-kill assay. As shown in Figure 3, both compound **8b** and CIP were bacteriostatic, not bactericidal, at 1 MIC. At 2 MIC, compound **8b** was rapidly bactericidal after 6 h and remained effective for 12 h. In contrast, CIP significantly inhibited bacterial reproduction but did not kill bacteria at 2 MIC. At 4 MIC, compound **8b** and CIP were rapidly bactericidal after 4 h and 6 h, respectively. The above results indicated the superiority of compound **8b** over the parent drug CIP in killing MRSA bacteria.

### 2.3. Cytotoxic Activity

Compounds **3a**, **8a**, and **8b** were also chosen to evaluate their cytotoxic activity against one human normal cell lines: HEK 293T. No effect of compounds **3a**, **8a**, and **8b** on cell proliferation was found at a concentration of 20 μM. Due to the poor solubility of the compounds, we can’t obtain their accurate IC_50_ values at higher test concentration. Nonetheless, it can be seen that these compounds have no cytotoxicity at their antibacterial effective dose. 

### 2.4. Molecular Docking and Drug-like Properties Prediction

As we know, CIP targets bacterial type II topoisomerases, generally DNA gyrase and DNA topoisomerase IV, to inhibit the replication of DNA and then kill bacteria. Some studies have reported the crystal structure of CIP in a complex with DNA gyrase [32,33]. The key structural units such as 4-oxo-3-carboxylic acid, cyclopropyl, piperazinyl, and fluoro moiety in CIP have been elucidated for their crucial interactions with the enzymes. To gain a deeper understanding of the molecular interactions between the synthesized hybrids and DNA gyrase, further investigation was conducted.

**Figure 4:**
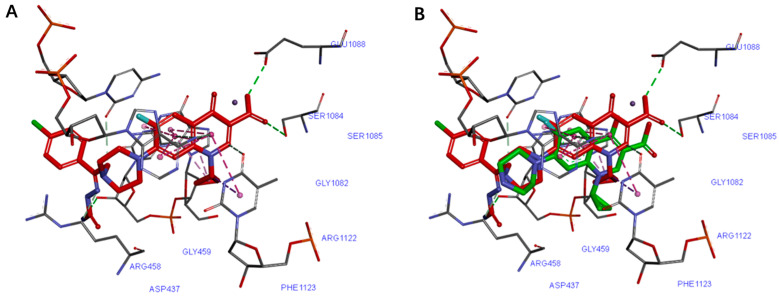
The binding pattern of **8b** with DNA gyrase (**A**), and the overlying of **8b** and CIP in the binding pocket (**B**).

To obtain a revealing insight into the molecular interactions of the synthesized hybrids with DNA gyrase, the co-binding pattern of **8b** complexed with *Staphylococcus aureus* DNA gyrase was virtually predicted and analyzed. As shown in Figure 4, the CIP unit of compound **8b** is located at the bottom of the active cavity and closely combined with the surrounding residues (Figure 4A).

The 4-oxo-3-carboxylic acid in the CIP unit formed a hydrogen bond with GLU1088 and SER1084 residues. The fluorine atom and piperazine groups in the CIP unit also interacted with corresponding amino acids. A hydrogen bonding between the semicarbazide unit and ARG458 residue further enhanced the binding force between molecule **8b** and DNA gyrase. The binding pattern of compound **8b** with DNA gyrase was superimposed on the co-crystallized CIP (Figure 4B), which gave an intuitive presentation that **8b** and the parent drug CIP had a similar binding mode with DNA gyrase.

Lipinski’s “Rule of Five” is widely used in early drug development, such as drug design and screening. It suggests that most “drug-like” molecules have similar parameters, including LogP ≤ 5, molecular weight (MW) ≤ 500, the number of H-bond acceptors (HAB) ≤ 10, the number of H-bond donors (HBD) ≤ 5, and the number of rotatable bonds (ROTB) ≤ 10. As listed in Table 3, the majority of CIP hybrids exhibited one Lipinski’s violation, which indicates that they have good drug-likeliness. Because of the high molecular weight of CIP, its hybrid compounds mostly exceed MW 500. However, considering the good bioavailability of CIP, the bioavailability of its hybrids was supposed to be not bad.

## 3. Materials and Methods

### 3.1. Chemical Part

The NMR spectra were measured on a Bruker NMR spectrometer (Bruker, Fallanden, Switzerland). High-resolution mass spectra were determined on a Bruker MALDI-TOF/TOF mass spectrometer (Bruker Daltonics, Bremen, Germany).

#### 3.1.1. Synthesis Procedure of 1-(3-Bromopropyl)-1*H*-indole-3-carbaldehyde (**1**)

3-indole formaldehyde (2.90 g, 0.02 mol) and NaH (0.48 g, 0.02 mol) were mixed in a 50 mL flask containing 10 mL of acetonitrile and heated up to reflux. After 1 h, 4.82 g (0.024 mol) of 1,3-dibromopropane was added dropwise, and the mixture was refluxed for 24 h. Evaporating the solvent, 30 mL of water was added into the residue, which was extracted with dichloromethane (30 mL × 3). The combined dichloromethane was washed with saturated salt water (30 mL × 2) twice. Then, the dichloromethane was dried with MgSO_4_ and purified on a silica gel column to obtain compound **1**. Red oil, yield: 35%. ^1^H-NMR (CDCl_3_, 400 MHz): *δ* 2.42 (t, *J* = 5.9 Hz, 2H, CH_2_), 3.36 (t, *J* = 5.6 Hz, 2H, BrCH_2_), 4.42 (t, *J* = 6.1 Hz, 2H, NCH_2_), 7.36–7.45 (m, 3H, Ph-H), 7.81 (s, 1H, NCH=C), 8.33 (d, *J* = 6.3 Hz, 1H, Ph-H), 10.03 (s, 1H, CHO).

#### 3.1.2. Synthesis Procedure of Ciprofloxacin–Indole Hybrid **2**

CIP (0.33 g, 0.001 mol), compound **1** (0.31 g, 0.0012 mol), and Na_2_CO_3_ (0.42 g, 0.004 mol) in acetonitrile (30 mL) were stirred and refluxed for 36 h. Evaporating the solvent, 30 mL of water was added followed by the 10% HCl to adjust the PH to 7. The precipitate was obtained via filtration and purified via silica gel column chromatography (CH_2_Cl_2_:CH_3_OH = 15:1) to give compound **2**. Light yellow solid, m.p. 207–210 °C, yield: 44%. ^1^H-NMR (DMSO-*d*_6_, 300 MHz): *δ* 1.14–1.23 (m, 4H, Cyclopropyl-H), 2.04 (t, 2H, *J* = 6.1 Hz, CH_2_), 2.35 (t, 2H, *J* = 5.9 Hz, BrCH_2_), 2.54 (s, 4H, NCH_2_), 3.31 (s, 4H, NCH_2_), 3.83 (s, 1H, NCH), 4.37 (t, *J* = 6.5 Hz, 2H, NCH_2_), 7.26 (t, *J* = 7.5 Hz, 1H, Ph-H), 7.33 (t, *J* = 7.6 Hz, 1H, Ph-H), 7.54 (d, 1H, *J* = 7.3 Hz, Ph-H), 7.68 (d, *J* = 8.2 Hz, 1H, Ph-H), 7.88 (d, 1H, *J* = 13.3 Hz, Ph-H), 8.11 (d, *J* = 7.8 Hz, 1H, Ph-H), 8.35 (s, 1H, Ph-H), 8.66 (s, 1H, PH-H), 9.93 (s, 1H, CHO), 15.20 (s, 1H, carboxyl). ^13^C-NMR (DMSO-*d*_6_, 75 MHz): *δ* 184.9, 176.8, 166.4, 153.5 (d, ^1^*J*_c-f_ = 247.6 Hz), 148.5, 145.6 (d, ^2^*J*_c-f_ = 10.5 Hz), 141.4, 139.6, 137.6, 125.1, 123.9, 122.9, 121.5, 119.1 (d, ^3^*J*_c-f_ = 7.5 Hz), 117.6, 111.6, 111.2, 107.2, 106.8 (d, ^4^*J*_c–f_ = 2.9 Hz), 54.7, 52.7, 49.8, 44.8, 36.3, 26.7, 8.0. ESI-HRMS calcd for C_29_H_30_FN_4_O_4_^+^ ([M + H]^+^): 517.2246; measured: 517.2250.

#### 3.1.3. Synthesis Procedure of Ciprofloxacin–Indole Hybrid **3a**

A mixture of compound **2** (0.052 g, 0.0001 mol), semicarbazide hydrochloride (0.033 g, 0.0003 mol), and CH_3_COONa (0.024 g, 0.0003 mol) in methanol (10 mL) was stirred and refluxed for 42 h. When the reaction was completed, 3 mL of water was added followed by concentrating the mixture to half its volume. The residue was put in the refrigerator overnight, and the precipitate formed was filtered and recrystallized with 50% alcohol to obtain compound **3a**. Yellow solid, m.p. 251–254 °C, yield: 53%. ^1^H-NMR (CDCl_3_, 500 MHz): *δ* 1.18–1.34 (m, 4H, Cyclopropyl-H), 2.31 (t, 2H, *J* = 6.4 Hz, NCH_2_), 2.51 (s, 4H, NCH_2_), 3.31 (s, 4H, NCH_2_), 3.63 (s, 1H, NCH), 3.76–3.87 (m, 2H, CH_2_), 4.34 (t, 2H, *J* = 6.4 Hz, NCH_2_), 6.20 (s, 2H, CONH_2_), 7.14–7.28 (m, 2H, Ph-H), 7.56–7.60 (m, 2H, Ph-H), 7.76–7.78 (m, 1H, Ph-H), 7.92–7.97 (d, 1H, Ph-H), 8.07 (s, 1H, Ph-H), 8.16–8.20 (m, 1H, Ph-H), 8.69 (d, 1H, *J* = 4.6 Hz, Ph-H), 9.91 (s, 1H, CONH), 15.08 (s, 1H, carboxyl). ^13^C-NMR (126 MHz, DMSO-*d_6_* + CDCl_3_) δ 176.9, 166.3, 157.3, 153.3(d, ^1^*J*_c-f_ = 248.5 Hz), 148.6, 144.0, 143.6, 139.5, 137.3, 137.2, 132.0, 125.1, 123.0, 122.6, 121.07, 116.9, 111.8, 111.6, 110.5, 107.5, 53.9, 51.2, 46.9, 43.6, 36.3, 29.5, 8.1. ESI-HRMS calcd for C_30_H_33_FN_7_O_4_^+^ ([M + H]^+^): 574.2573; measured: 574.2581. ESI-HRMS calcd for C_30_H_33_FN_7_O_4_^+^ ([M + H]^+^): 574.2573; measured: 574.2581.

#### 3.1.4. Synthesis Procedure of Ciprofloxacin–Indole Hybrid **3b**

A mixture of compound **2** (0.052 g, 0.1 mmol), thiosemicarbazone (0.027 g, 0.3 mmol), and CH_3_COONa (0.024 g, 0.3 mmol) in methanol (10 mL) were stirred and refluxed for 72 h. When the reaction was completed, 3 mL of water was added followed by concentrating the mixture to half its volume. The residue was put in the refrigerator overnight, and the precipitate formed was filtered and recrystallized with 50% alcohol to obtain compound **3b**. Yellow solid, m.p. 218–221 °C, yield: 52%. ^1^H-NMR (DMSO-*d_6_*, 300 MHz): *δ* 1.19–1.33 (m, 4H, Cyclopropyl-H), 2.00 (t, 2H, *J* = 6.5 Hz, NCH_2_CH_2_), 2.32 (t, 2H, *J* = 6.4 Hz, NCH_2_), 2.54 (s, 4H, NCH_2_), 3.31 (s, 4H, NCH_2_), 3.81 (s, 1H, NCH), 4.27 (t, 2H, *J* = 6.7 Hz, NCH_2_), 7.16 (t, 1H, *J* = 7.6 Hz, Ph-H), 7.26 (t, 1H, *J* = 7.6 Hz, Ph-H), 7.40 (s, 1H, Ph-H), 7.51–7.58 (m, 2H, Ph-H), 7.84 (d, 1H, *J* = 7.9 Hz, Ph-H), 7.87 (s, 1H, Ph-H), 8.01 (s, 1H, Ph-H), 8.22 (d, 1H, *J* = 7.9 Hz, Ph-H), 8.29 (s, 1H, Ph-H), 8.64 (s, 1H, Ph-H), 11.47 (s, 1H, NHCS), 15.18 (s, 1H, carboxyl). ^13^C-NMR (DMSO-*d_6_*, 75 MHz): *δ* 177.0, 176.8, 166.4, 153.5 (d, ^1^*J*_c-f_ = 248.0 Hz), 148.4, 145.6 (d, ^2^*J*_c-f_ = 9.9 Hz), 140.8, 139.6, 137.5, 134.3, 124.9, 123.1, 122.9, 121.3, 119.0 (d, ^3^*J*_c-f_ = 7.7 Hz), 111.4 (d, ^2^J_c-f_ = 23.2 Hz), 110.8, 110.7, 107.2, 106.7 (d, ^4^*J*_c-f_ = 2.7 Hz), 54.8, 52.7, 49.8, 44.2, 36.3, 27.0, 8.0. ESI-HRMS calcd for C_30_H_33_FN_7_O_3_S^+^ ([M + H]^+^): 590.2344; measured: 590.2355.

#### 3.1.5. Synthesis Procedure of Ciprofloxacin–Indole Hybrid **3c**

A mixture of compound **2** (0.052 g, 0.0001 mol), benzoyl hydrazide (0.04 g, 0.0003 mol), and CH_3_COONa (0.024 g, 0.0003 mol) in methanol (10 mL) was stirred and refluxed for 72 h. When the reaction was completed, 3 mL of water was added followed by concentrating the mixture to half its volume. The residue was put in the refrigerator overnight, and the precipitate formed was filtered and recrystallized with 50% alcohol to obtain compound **3c**. White solid, m.p. 249–252 °C, yield: 24%. ^1^H-NMR (DMSO-*d_6_*, 400 MHz): *δ* 1.18–1.34 (m, 4H, Cyclopropyl-H), 2.01 (t, 2H, *J* = 7.5 Hz, NCH_2_), 2.33 (t, 2H, *J* = 6.9 Hz, NCH_2_), 2.54 (s, 4H, NCH_2_), 3.31 (s, 4H, NCH_2_), 3.84 (s, 1H, NCH), 4.30 (t, 2H, *J* = 6.9 Hz, NCH_2_), 7.20 (t, 1H, *J* = 7.5 Hz, Ph-H), 7.28 (t, 1H, *J* = 7.6 Hz, Ph-H), 7.50–7.60 (m, 5H, Ph-H), 7.81–7.93 (m, 4H, Ph-H), 8.32 (d, 1H, *J* = 7.8 Hz, Ph-H), 8.64 (s, 1H, Ph-H), 11.56 (s, 1H, CONH), 15.19 (s, 1H, carboxyl). ^13^C-NMR (DMSO-*d_6_*, 101 MHz): *δ* 176.7, 166.5, 162.9, 153.5 (d, ^1^*J*_c-f_ = 249.6 Hz), 148.4, 144.9, 139.6, 137.5, 134.5, 133.6, 133.6, 131.8, 128.8, 128.7, 127.9, 127.4, 125.3, 123.1, 122.7, 121.0, 111.5, 111.4, 110.8, 106.7, 54.9, 52.7, 49.9, 44.2, 36.3, 27.1, 8.0. ESI-HRMS calcd for C_36_H_36_FN_6_O_4_^+^ ([M + H]^+^): 635.2777; measured: 635.2787.

#### 3.1.6. Synthesis Procedure of Ciprofloxacin–Indole Hybrid **3d**

Compound **2** (0.052 g, 0.0001 mol), methoxyamine hydrochloride (0.024 g, 0.0003 mol), and CH_3_COONa (0.024 g, 0.0003 mol) in methanol (10 mL) was stirred and refluxed for 72 h. Then 3 mL of water was added followed by concentrating the mixture to half its volume. The residue was cooled at 4 °C overnight, and the solid formed was filteredand recrystallized with 50% alcohol to obtain compound **3d**. White solid, m.p. 225–228 °C, yield: 56%. ^1^H-NMR (DMSO-*d_6_*, 400 MHz): *δ* 1.17–1.35 (m, 4H, Cyclopropyl-H), 2.34 (s, 2H, NCH_2_), 3.23–3.35 (m, 8H, NCH2), 3.63 (s, 2H, CH_2_), 3.88 (s, 4H, NCH, OCH_3_), 4.38 (t, *J* = 6.8 Hz, 2H, NCH_2_), 7.18 (t, 1H, *J* = 7.5 Hz, Ph-H), 7.27 (t, *J* = 7.6 Hz, 1H, Ph-H), 7.57–7.67 (m, 2H, Ph-H), 7.80–8.03 (m, 3H, Ph-H), 8.29 (d, *J* = 7.8 Hz, 1H, Ph-H), 8.66 (s, 1H, CH=N), 15.12 (s, 1H, carboxyl). ^13^C-NMR (DMSO-*d_6_*, 101 MHz): *δ* 176.8, 166.3, 153.3 (d, ^1^*J*_c-f_ = 250.3 Hz), 148.6, 145.1, 139.5, 138.9, 137.2, 135.3, 127.3, 125.1, 123.2, 122.8, 122.3, 121.1 (d, ^3^*J*_c-f_ = 12.6 Hz), 119.1, 111.6 (d, ^2^*J*_c-f_ = 22.3 Hz), 110.9 (d, ^4^*J*_c-f_ = 3.5 Hz), 107.3, 62.2, 61.6, 55.4, 51.0, 46.7, 43.8, 36.4, 8.1. ESI-HRMS calcd for C_30_H_33_FN_5_O_4_^+^ ([M + H]^+^): 546.2511; measured: 546.2515.

#### 3.1.7. Synthesis Procedure of 1-(4-Bromobutyl)-1*H*-indole-3-carbaldehyde (**4a**)

3-indole formaldehyde (1.45 g, 0.01 mol) and NaH (0.24 g, 0.01 mol) were mixed in a 50 mL of flask containing 10 mL of acetonitrile and heated up to reflux. After 1 h, 2.41 g (0.012 mol) of 1,3-dibromopropane was put into dropwise, and the mixture was refluxed for 24 h. Evaporating the solvent, 30 mL of water was added and extracted with dichloromethane three times (30 mL × 3). The organic layer was washed with saturated salt water (30 mL × 2) twice. The organic layer was dried with MgSO_4_ and purified on a silica gel column (PE: EA = 4:1) to obtain compound **1**. Red oil, yield: 36%. ^1^H-NMR (CDCl_3_, 400 MHz): *δ* 2.44 (t, *J* = 5.9 Hz, 2H, CH_2_), 3.36 (t, *J* = 5.6 Hz, 2H, BrCH_2_), 4.42 (t, *J* = 6.1 Hz, 2H, NCH_2_), 7.36–7.45 (m, Ph-H, 3H), 7.81 (s, 1H, NCH=C), 8.33 (d, *J* = 6.3 Hz, 1H, Ph-H), 10.03 (s, 1H, CHO).

#### 3.1.8. Synthesis Procedure of 1-(5-Bromopentyl)-1*H*-indole-3-carbaldehyde (**4b**)

Compound **4b** was synthesized using the same procedure as compound **4a**. Brown oil, yield: 78%. ^1^H-NMR (CDCl_3_, 500 MHz): *δ* 1.49–1.55 (m, 2H, CH_2_), 1.86–1.96 (m, 4H, CH_2_), 3.38 (t, 2H, *J* = 6.6 Hz, BrCH_2_), 4.20 (t, 2H, *J* = 7.1 Hz, NCH_2_), 7.30–7.39 (m, 3H, Ph-H), 7.72 (s, 1H, NCH=C), 8.31 (d, 1H, *J* = 6. Hz, Ph-H), 10.00 (s, 1H, CHO). ^13^C-NMR (CDCl_3_, 126 MHz): *δ* 183.4, 137.1, 136.1, 124.5, 123.0, 121.9, 121.2, 117.1, 109.0, 46.1, 32.1, 31.0, 27.9, 24.4.

#### 3.1.9. Synthesis Procedure of Ciprofloxacin–Indole Hybrid **5a**

CIP (0.33 g, 1 mmol), compound **4a** (0.44 g, 1.2 mmol), and Na_2_CO_3_ (0.42 g, 4 mol) in CH_3_CN (30 mL) were stirred and refluxed for 24 h. Evaporating the solvent, 30 mL of water was poured into the flask, followed by 10% HCl to balance the PH to 7. The liquid was extracted using dichloromethane (30 mL × 3). The combined CH_2_Cl_2_ layer was dried using MgSO_4_ and purified on column chromatography with silica gel separation (dichloromethane: methanol = 30:1) to obtain compound **5a**. Yellow solid, M.p. 214–215 °C, yield: 47%. ^1^H-NMR (DMSO-*d_6_*, 300 MHz): *δ* 1.30–1.49 (m, 6H, Cyclopropyl-H, CH_2_), 1.84–1.94 (m, 2H, CH_2_), 2.39 (t, 2H, *J* = 6.8 Hz, NCH_2_), 2.55 (s, 4H, NCH_2_), 3.39 (s, 4H, NCH_2_), 3.82 (s, 1H, NCH), 4.30 (t, 2H, *J* = 6.7 Hz, NCH_2_), 7.24–7.36 (m, Ph-H, 2H), 7.54 (d, *J* = 7.6 Hz, 1H, Ph-H), 7.68 (d, *J* = 8.1 Hz, 1H, Ph-H), 7.90 (d, *J* = 13.4 Hz, 1H, Ph-H), 8.12 (d, *J* = 7.9 Hz, 1H, Ph-H), 8.35 (s, 1H, Ph-H), 8.66 (s, 1H, PH-H), 9.93 (s, 1H, CHO), 15.20 (s, 1H, carboxyl). ^13^C-NMR (75 MHz, DMSO-*d*_6_) δ 184.9, 176.6, 166.4, 155.1, 148.5, 141.2, 139.6, 137.5, 125.2, 123.9, 123.6, 123.2, 122.9, 121.5, 117.5, 111.6, 111.5, 107.2, 106.8, 57.2, 52.7, 49.9, 46.7, 36.3, 32.0, 29.9, 29.5, 27.6, 23.7, 8.0. ESI-HRMS calcd for C_30_H_34_FN_4_O_4_^+^ ([M + H]^+^): 531.2402; measured: 531.2411.

#### 3.1.10. Synthesis Procedure of Ciprofloxacin–Indole Hybrid **5b**

Compound **5b** was obtained using the same method as compound **5a**. Light yellow solid, M.p. 243–245 °C, yield: 44%. ^1^H-NMR (CDCl_3_, 400 MHz): *δ* 1.17–1.21 (m, 2H, CH_2_), 1.39–1.43 (m, 4H, Cyclopropyl-H), 1.54–1.61 (m, 2H, CH_2_), 1.92–2.00 (m, 2H, CH_2_), 2.40 (t, 2H, *J* = 6.8 Hz, NCH_2_), 2.61 (s, 4H, NCH_2_), 3.29 (s, 4H, NCH_2_), 3.58 (s, 1H, NCH), 4.23 (t, 2H, *J* = 7.1 Hz, NCH_2_), 7.29–7.41 (m, 4H, Ph-H), 7.75 (s, 1H, Ph-H), 7.97 (d, 1H, *J* = 13.0 Hz, Ph-H), 8.28 (d, 1H, *J* = 7.4 Hz, Ph-H), 8.74 (s, 1H, Ph-H), 10.00 (s, 1H, CHO), 15.06 (s, 1H, carboxyl). ^13^C-NMR (CDCl_3_, 101 MHz): *δ* 184.5, 177.1, 167.1, 153.7 (d, ^1^*J*_c-f_ = 252.7 Hz), 147.4, 145.9 (d, ^3^*J*_c-f_ = 10.3 Hz), 139.1, 138.2, 137.2, 125.5, 124.0, 122.9, 122.1, 119.8 (d, ^3^*J*_c-f_ = 7.9 Hz), 118.0, 112.3 (d, ^2^*J*_c-f_ = 23.4 Hz), 110.1, 108.1, 104.9 (d, ^4^*J*_c-f_ = 3.6 Hz), 57.8, 53.5, 52.8, 49.7 (d, ^r^*J*_c-f_ = 4.9 Hz), 47.2, 35.3, 29.7, 29.4, 26.2, 24.5, 8.2. ESI-HRMS calcd for C_31_H_36_FN_4_O_4_^+^ ([M + H]^+^): 545.2559; measured: 545.2567.

#### 3.1.11. Synthesis Procedure of Ciprofloxacin–Indole Hybrid **6a**

Compound **5a** (0.195 g, 0.37 mmol), semicarbazide hydrochloride (0.123 g, 1.10 mmol), and CH_3_COONa (0.09 g, 1.10 mmol), and methanol (15 mL) were stirred and heated for 24 h. The solvent was concentrated to half its volume, and 8 mL of water was added. The flask was put in the refrigerator overnight, and the precipitate formed was filtered to obtain compound **6a**. Yellow solid, M.p. 203–204 °C, yield: 91%. ^1^H-NMR (DMSO-*d_6_*, 300 MHz): *δ* 1.19–1.37 (m, 4H, Cyclopropyl-H), 1.73–1.91 (m, 4H, CH_2_), 2.58 (s, 4H, NCH_2_), 3.16 (s, 4H, NCH_2_), 3.51 (t, 2H, *J* = 7.3 Hz, NCH_2_), 3.85 (s, 1H, NCH), 4.26 (t, 2H, *J* = 7.0 Hz, NCH_2_), 6.16 (s, 2H, CONH_2_), 7.13–7.28 (m, 2H, Ph-H), 7.54–7.61 (m, 2H, Ph-H), 7.79 (s, 1H, Ph-H), 7.95 (d, 1H, *J* = 13.1 Hz, Ph-H), 8.08 (s, 1H, Ph-H), 8.17 (d, 1H, *J* = 8.1 Hz, Ph-H), 8.68 (s, 1H, Ph-H), 9.84 (s, 1H, CONH), 15.06 (s, 1H, carboxyl). ^13^C-NMR (DMSO-*d*_6_, 75 MHz): *δ* 176.8, 166.3, 157.3, 153.3 (d, ^1^*J*_c-f_ = 247.7 Hz), 148.7, 144.3 (d, ^3^*J*_c-f_ = 7.8 Hz), 139.5, 137.4, 137.1, 132.3, 125.0, 123.0, 122.6, 121.0, 119.8, (d, ^3^*J*_c-f_ = 4.9 Hz), 111.7 (d, ^2^*J*_c-f_ = 22.3 Hz), 111.5, 110.6, 107.4, 107.3, 55.5, 50.9, 46.8, 45.5, 36.4, 27.2, 21.1, 8.1. ESI-HRMS calcd for C_31_H_37_FN_7_O_4_^+^ ([M + H]^+^): 588.2729; measured: 588.2729.

#### 3.1.12. Synthesis Procedure of Ciprofloxacin–Indole Hybrid **6b**

Compound **6b** was obtained from the same procedure as compound **6a**. Yellow solid, M.p. 205–207 °C, yield: 91%. ^1^H-NMR (CDCl_3_, 400 MHz): *δ* 1.07–1.11 (m, 2H, CH_2_), 1.29–1.42 (m, 4H, Cyclopropyl-H), 1.49–1.56 (m, 2H, CH_2_), 1.87–1.94 (m, 2H, CH_2_), 2.35 (t, 2H, *J* = 6.9 Hz, NCH_2_), 2.50 (s, 4H, NCH_2_), 3.21 (s, 4H, NCH2), 3.48 (s, 1H, NCH), 4.19 (t, 2H, *J* = 6.6 Hz, NCH_2_), 6.17 (s, 2H, CONH_2_), 7.18–7.42 (m, 6H, Ph-H), 7.90 (d, 1H, *J* = 12.9 Hz, Ph-H), 8.21 (s, 1H, *J* = 7.8 Hz, Ph-H), 8.41 (s, 1H, Ph-H), 8.69 (s, 1H, CONH), 15.10 (s, 1H, carboxyl). ^13^C-NMR (CDCl_3_,101 MHz): *δ* 176.9, 167.1, 154.4, 153.7 (d, ^1^*J*_c-f_ = 252.7 Hz), 147.0, 146.0 (d, ^3^*J*_c-f_ = 11.0 Hz), 138.9, 137.1, 132.2, 125.9, 123.1, 122.1, 122.1, 121.1, 119.53 (d, ^3^*J*_c-f_ = 7.5 Hz), 112.0 (d, ^2^*J*_c-f_ = 23.1 Hz), 111.6, 110.0, 107.7, 105.0 (d, ^4^*J*_c-f_ = 2.6 Hz), 56.8, 52.6, 49.7, 49.7, 46.6, 35.2, 29.7, 28.8, 25.7, 23.8, 8.1. ESI-HRMS calcd for C_32_H_39_FN_7_O_4_^+^ ([M + H]^+^): 602.2886; measured: 602.2886.

#### 3.1.13. Synthesis Procedure of Ciprofloxacin–Acetophenone Hybrid **7a**

A mixture of CIP (0.66 g, 0.002 mol), α-Bromo-4-methoxyacetophenone (0.55 g, 0.0024 mol), and NaHCO_3_ (0.20 g, 0.0024 mol) in DMF (10 mL) was stirred at room temperature for 17 h. The mixture was filtered and dried to give the crude product, which was recrystallized in alcohol to obtain compound **7a**. Light yellow solid, M.p. 128–129 °C, yield: 83%. ^1^H-NMR (DMSO-*d_6_*, 400 MHz): *δ* 1.17–1.34 (m, 4H, Cyclopropyl-H), 2.75 (s, 4H, NCH_2_), 2.90 (s, 1H, NCH), 3.33 (s, 4H, NCH_2_), 3.86 (s, 3H, OCH_3_), 3.89 (s, 2H, COCH_2_N), 7.05 (d, 2H, *J* = 7.8 Hz, Ph-H), 7.59 (d, 1H, *J* = 6.7 Hz, Ph-H), 7.90–7.96 (m, 1H, Ph-H), 8.02 (d, 2H, *J* = 7.9 Hz, Ph-H), 8.67 (s, 1H, Ph-H), 15.22 (s, 1H, carboxyl). ESI-HRMS calcd for C_26_H_27_FN_3_O_5_^+^ ([M + H]^+^): 480.1929; measured: 480.1937.

#### 3.1.14. Synthesis Procedure of Ciprofloxacin–Acetophenone Hybrid **7b**

Compound **7b** was obtained from the same procedure as compound **7a**. Light yellow solid, M.p. 207–208 °C, yield: 76%. ^1^H-NMR (DMSO-*d_6_*, 500 MHz): *δ* 1.17–1.32 (m, 4H, Cyclopropyl-H), 2.76 (t, 4H, *J* = 4.9 Hz, NCH_2_), 3.35 (t, 4H, *J* = 4.9 Hz, NCH_2_), 3.82 (s, 1H, NCH), 3.96 (s, 2H, COCH_2_N), 7.57 (d, 1H, *J* = 7.4 Hz, Ph-H), 7.61 (d, 2H, *J* = 8.6 Hz, Ph-H), 7.91(d, 1H, *J* = 13.2 Hz, Ph-H), 8.04 (d, 2H, *J* = 8.6 Hz, Ph-H), 8.66 (s, 1H, Ph-H), 15.21 (s, 1H, carboxyl). ESI-HRMS calcd for C_25_H_24_ClFN_3_O_4_^+^ ([M + H]^+^): 484.1434; measured: 484.1447.

#### 3.1.15. Synthesis Procedure of Ciprofloxacin–Acetophenone Hybrid **8a**

A mixture of compound **7a** (0.177 g, 0.37 mmol), semicarbazide hydrochloride (0.123 g, 1.10 mmol), and CH_3_COONa (0.09 g, 1.10 mmol) in methanol (15 mL) were stirred and refluxed for 15 h. The solvent was concentrated to half its volume, and 8 mL of water was poured into the flask. The flask was kept in the refrigerator for 12 h, and the solid formed was filtered to obtain hybrid **8a**. Light yellow solid, M.p. 244–246 °C, yield: 90%. ^1^H-NMR (DMSO-*d_6_*, 500 MHz): *δ* 1.18–1.32 (m, 4H, Cyclopropyl-H), 2.71 (s, 4H, NCH_2_), 3.34 (s, 4H, NCH_2_), 3.79 (s, 5H, COCH_2_N, OCH_3_), 3.85 (s, 1H, NCH), 6.54 (s, 2H, CONH_2_), 6.93 (d, 2H, *J* = 8.2 Hz, Ph-H), 7.60 (d, 1H, *J* = 7.1 Hz, Ph-H), 7.86 (d, 2H, *J* = 8.2 Hz, Ph-H), 7.91 (d, *J* = 13.1 Hz, 1H, Ph-H), 8.66 (s, 1H, Ph-H), 10.39 (s, 1H, CONH), 15.19 (s, 1H, carboxyl). ^13^C-NMR (DMSO-*d*_6_, 126 MHz): *δ* 176.8, 166.5, 157.0, 153.4 (d, ^1^*J*_c-f_ = 252.3 Hz), 148.4, 145.5 (d, ^3^*J*_c-f_ = 10.5 Hz), 142.7, 139.6, 138.0, 129.0, 128.7, 126.7, 119.3 (d, ^3^*J*_c-f_ = 8.8 Hz), 111.4 (d, ^2^*J*_c-f_ = 22.4 Hz), 107.2, 107.1 (d, ^4^*J*_c-f_ = 3.3 Hz), 55.1, 55.1, 52.0, 49.9 (d, ^r^*J*_c-f_ = 4.5 Hz), 36.4, 8.0. ESI-HRMS calcd for C_27_H_30_FN_6_O_5_^+^ ([M + H]^+^): 537.2256; measured: 537.2264.

#### 3.1.16. Synthesis Procedure of Ciprofloxacin–Acetophenone Hybrid **8b**

Compound **8b** was obtained using the same procedure as compound **8a**. Yellow solid, M.p. 252–253 °C, yield: 79%. ^1^H-NMR (DMSO-*d*_6_, 400 MHz): *δ* 1.17–1.33 (m, 4H, Cyclopropyl-H), 2.71 (s, 4H, NCH_2_), 3.33 (s, 5H, NCH_2_ and NCH), 3.81 (s, 2H, COCH_2_N), 6.68 (s, 2H, CONH_2_), 7.41 (d, 2H, *J* = 8.5 Hz, Ph-H), 7.57 (d, 1H, *J* = 7.1 Hz, Ph-H), 7.88 (d, 1H, *J* = 13.0 Hz, Ph-H), 7.96 (d, 2H, *J* = 8.5 Hz, Ph-H), 8.64 (s, 1H, Ph-H), 10.53 (s, 1H, CONH), 15.18 (s, 1H, carboxyl). ^13^C-NMR (DMSO-*d*_6_, 101 MHz): *δ* 176.8, 166.5, 157.0, 153.4 (d, ^1^*J*_c-f_ = 251.1 Hz), 148.4, 145.5 (d, ^3^*J*_c-f_ = 10.2 Hz), 141.5, 139.6, 136.8, 133.7, 128.7, 128.5, 119.2 (d, ^3^*J*_c-f_ = 8.6 Hz), 111.4 (d, ^2^*J*_c-f_ = 22.8 Hz), 107.2, 107.1 (d, ^4^*J*_c-f_ = 3.2 Hz), 54.6, 52.0, 49.9 (d, ^r^*J*_c-f_ = 4.9 Hz), 36.3, 8.0. ESI-HRMS calcd for C_26_H_27_ClFN_6_O_4_^+^ ([M + H]^+^): 541.1761; measured: 541.1760.

### 3.2. Pharmacological Assays

#### 3.2.1. Antibacterial Activity Evaluation

The antibacterial activity in vitro was investigated using a two-fold serial dilution technique, and the final concentrations of samples tested were in the range of 0.625–32 μg/mL. The test bacteria were cultured in Tryptone Soya Broth (TSB) or Mueller–Hinton broth (MHB) until they reached the mid-log phase. The cultures were then diluted 1000-fold in the same medium. Bacteria at a concentration of 10^5^ CFU/mL were inoculated into MHB or TSB and dispensed at 0.2 mL per well into a 96-well microtiter plate. CIP, norfloxacin, and penicillin were used as the positive controls. The test compounds were prepared in DMSO with a final concentration not exceeding 0.05%. The minimum inhibitory concentration (MIC) was determined as the concentration of the test sample that inhibited more than 90% of bacterial reproduction after 22 h of incubation at 37 °C. Bacterial growth was evaluated by record the absorbance at 630 nm on a microplate reader [34].

#### 3.2.2. Propensity Evaluation for the Development of Bacterial Resistance

Compound **8b** and antibiotic control ciprofloxacin (CIP) were selected to evaluate the propensity for developing bacterial resistance. First, the MIC values of **8b** and CIP against *S. aureus* CMCC 25923 and *E. coli* CMCC 44568 were determined. Subsequently, the bacteria were cultured at the sub-MIC (MIC/2) concentration of compound **8b** and CIP. One part was transferred to a new culture tube and continued to be treated with MIC/2 drugs. At the same time, another part was taken to determine the new MIC value of compound **8b** and CIP. The process was repeated for 20 generations for each strain. A time curve was drawn for the MIC value of compound **8b** and CIP in days (passage times). As the number of generations increases, if the MIC value of the drug increases by more than four times compared to the initial value, it indicates that the drug has a tendency to develop bacterial resistance.

#### 3.2.3. Time-Kill Assay

To investigate the time-kill kinetics, methicillin-resistant *S. aureus* ATCC 33591 cultivated in MHB was utilized. Bacterial suspensions with a concentration of 10^5^ CFU/mL—containing compound **8b** or CIP at 1 MIC, 2 MIC, and 4 MIC (final concentration)—were subjected to incubation at 37 °C with agitation. At specific time intervals (0, 0.5, 1, 2, 3, 4, 6, 8, and 12 h), samples of the broth containing **8b** or CIP were collected. These samples were then serially diluted 1000-fold in a nutrient solution and plated onto sterile Mueller–Hinton agar medium. Subsequently, the plates were incubated at 37 °C for 24 h, and the resulting colony-forming units (CFU) were enumerated. Finally, the total bacterial count was obtained (log^10^ CFU/mL).

### 3.3. Evaluation of Cytotoxicity Activity In Vitro

The cytotoxicity experiment was conducted according to our previous publication [35].

### 3.4. Molecular Docking and Drug-like Properties Prediction

To investigate the interaction of hybrid **8b** with topoisomerase II DNA gyrase enzyme, the crystal structure was downloaded from RCSB PDB (PDB ID: 2XCT) and the molecular docking was performed using the Discovery Studio 2019. The 3D structure of compound **8b** was generated using Chemdraw12.0 software and then energetically optimized using Discovery Studio. For protein preparation, hydrogen atoms were added and water and impurities were eliminated. The original ligand cavity was defined as the binding active site. During the molecular docking process, the **8b** was copied into the binding active site. The docking interactions between the proteins and **8b** were analyzed and ranked, and the interaction pattern with the highest binding energy was selected for further analysis. The calculate molecular properties module of DS 2019 was used to predict the drug-like properties (i.e., MW, RotB, CLogP, nHBD, and nHBA) of the target compounds.

## 4. Conclusions

In summary, several CIP-indole and CIP-acetophenone hybrids were prepared and assessed for their antibacterial activities in vitro. All hybrid compounds displayed significant inhibitory activity against the tested strains, with hybrid **8b** showing the highest potency. In fact, hybrid **8b** exhibited greater inhibitory activity against *S. aureus* CMCC 25923 compared to the parent compound CIP. Moreover, the low drug resistance of **8b** and its superior bactericidal ability against CIP prompt us to conduct further research on it.

## Figures and Tables

**Table 1:** Antibacterial activity (MIC, μg/mL) of compounds **2**, **3a–d**, **5a–b**, **6a–b**, **7a–b**, and **8a–b** against gram-positive bacteria and gram-negative bacteria.

Compound		Gram-Positive Strains					Gram-Negative Strains				
	26003 *^a^*	6538 *^b^*	25923 *^c^*	336931 *^d^*	29212 *^e^*	63501 *^f^*	25922 *^g^*	44568 *^h^*	44103 *^i^*	27853 *^j^*	10104 *^k^*
**2**	4	0.5	0.5	4	0.5	0.5	8	0.5	0.5	8	0.5
**3a**	4	0.25	0.25	4	0.5	1	8	0.25	0.25	4	0.5
**3b**	4	4	4	16	32	2	16	8	4	8	8
**3c**	4	4	2	4	32	2	16	2	2	8	4
**3d**	4	2	1	4	8	1	16	1	1	8	2
**5a**	2	1	0.5	8	1	1	4	1	1	4	2
**5b**	16	4	1	16	4	16	32	2	2	16	8
**6a**	8	0.5	0.5	8	1	4	16	0.5	0.5	8	2
**6b**	8	2	0.5	16	8	8	16	1	4	16	16
**7a**	2	1	0.5	2	1	1	2	2	2	8	4
**7b**	0.5	0.5	0.125	1	0.5	0.5	1	0.5	0.5	2	1
**8a**	0.5	0.5	0.25	2	0.25	1	1	0.25	0.5	8	2
**8b**	0.25	0.25	0.0625	0.5	0.25	0.25	0.5	0.25	0.125	1	0.5
Ciprofloxacin	0.25	0.25	0.25	0.25	0.25	0.25	0.25	0.25	0.125	1	0.5
Norfloxacin	0.5	0.5	0.5	16	1	2	2	0.5	0.5	2	4
Penicillin	0.5	2	0.5	2	>32	>32	>32	>32	>32	>32	32

*^a^ Staphylococcus aureus* CMCC(B) 26003; *^b^ Staphylococcus aureus* ATCC 6538; *^c^ Staphylococcus aureus* CMCC 25923; *^d^ Streptococcus mutans* BNCC 336931; *^e^ Enterococcus faecalis* CMCC 29212; *^f^ Bacillus subtilis* CMCC 63501*; ^g^ Escherichia coli* CMCC 25922; *^h^ Escherichia coli* CMCC 44568; ^*i*^ *Escherichia coli CMCC* 44103; *^j^ Pseudomonas aeruginosa* CMCC 27853; ^*k*^ *Pseudomonas aeruginosa* CMCC 10104.

**Table 2:** Inhibitory effects (MIC, μg/mL) of hybrids **2**, **3a–d**, **5a–b**, **6a–b**, **7a–b**, and **8a–b** against clinical isolates of MDR strains.

Compounds	MDR Gram-Positive Strains		MDR Gram-Negative Strains
	43300 *^a^*	33591 *^b^*	BAA-196 *^c^*
**2**	2	2	16
**3a**	0.5	0.5	4
**3b**	16	8	32
**3c**	16	4	32
**3d**	8	4	16
**5a**	1	2	8
**5b**	4	4	32
**6a**	8	4	32
**6b**	4	16	16
**7a**	1	1	1
**7b**	0.5	0.5	1
**8a**	0.5	1	0.5
**8b**	0.25	0.25	0.25
Ciprofloxacin	0.25	0.25	0.25
Norfloxacin	0.5	0.5	0.5
Penicillin	32	>32	>32

*^a^ S. aureus* ATCC 43300; *^b^ S. aureus* ATCC 33591; *^c^* multidrug-resistant *E. coli* ATCC BAA-196.

**Table 3:** Drug-like property parameters of the hybrids **2**, **3a–d**, **5a–b**, **6a–b**, **7a–b**, and **8a–b**.

Compoud	MW	CLogP	HBD	HBA	n-ROTB	Lipinski’s Violation
**Rule**	≤500	≤5	≤5	<10	≤10	≤1
**2**	516.57	1.80	1	8	8	1
**3a**	573.63	1.41	4	11	9	2
**3b**	589.70	1.95	4	10	10	1
**3c**	634.71	3.17	2	10	10	1
**3d**	545.62	1.80	1	9	9	1
**5a**	530.60	2.07	1	8	9	1
**5b**	544.63	2.67	1	8	10	1
**6a**	587.66	1.68	4	11	10	2
**6b**	601.18	2.19	4	11	11	3
**7a**	479.51	1.10	1	8	7	0
**7b**	483.93	1.72	1	7	6	0
**8a**	536.56	0.52	4	11	8	2
**8b**	540.98	1.14	4	10	7	1

## Data Availability

Not applicable.

## Supplementary Materials

The following supporting information can be downloaded at: https://www.mdpi.com/article/10.3390/molecules28176325/s1.

## Sample Availability

Not available.
