# Supplementary material for: Design, Synthesis, and Antimicrobial Activity Evaluation of Ciprofloxacin—Indole Hybrids
Source: Molecules. 2023 Aug 29;28(17):6325. doi: 10.3390/molecules28176325 (PMC10488977; doi:10.3390/molecules28176325)
Supplement: Supplementary file 1 [file molecules-28-06325-s001.zip › molecules-2566789-SI.pdf]

Supporting Information

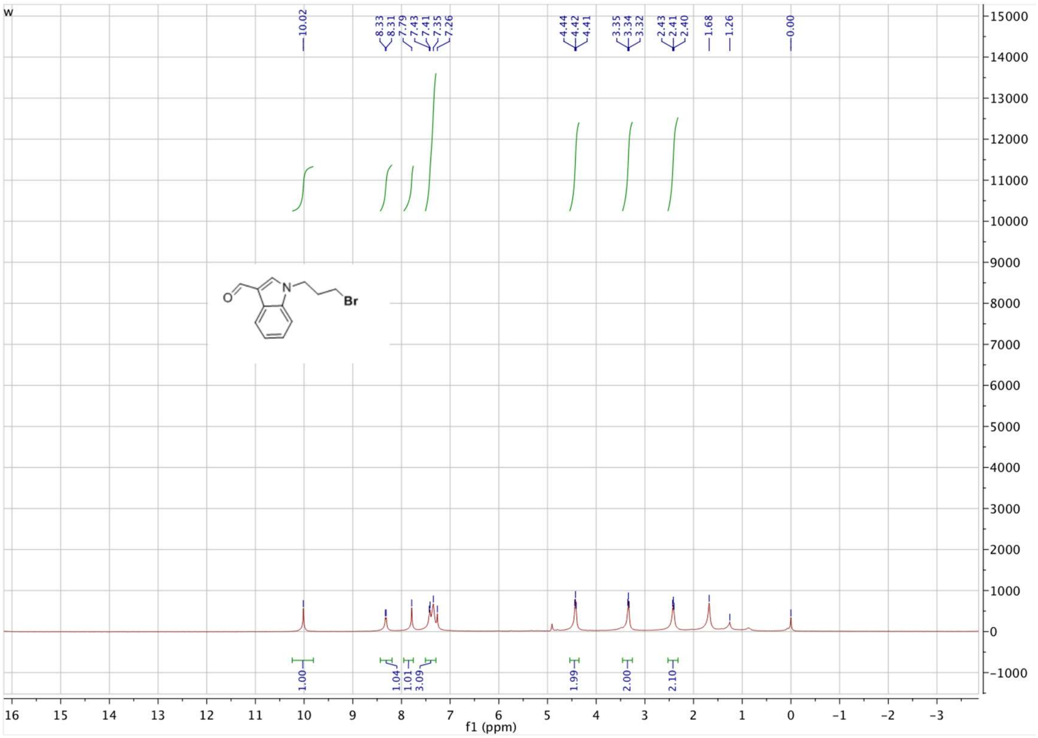

*^{1}H NMR of compound **1***

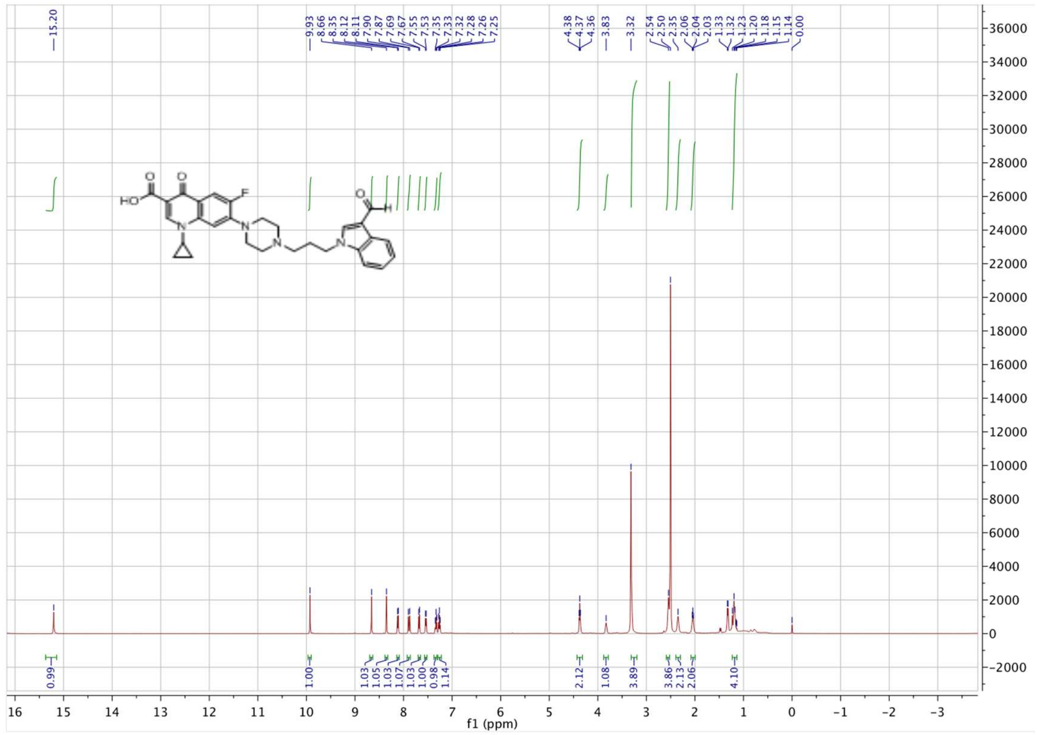

*^{1}H NMR of compound **2***

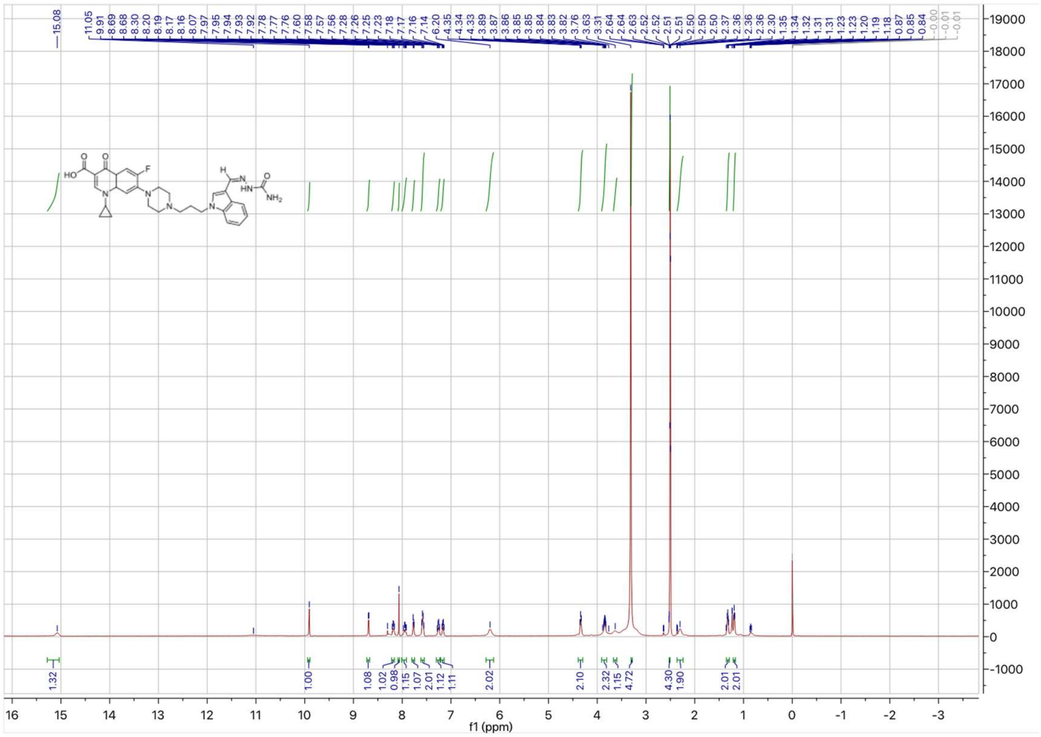

^{1}H NMR of compound ***3a***

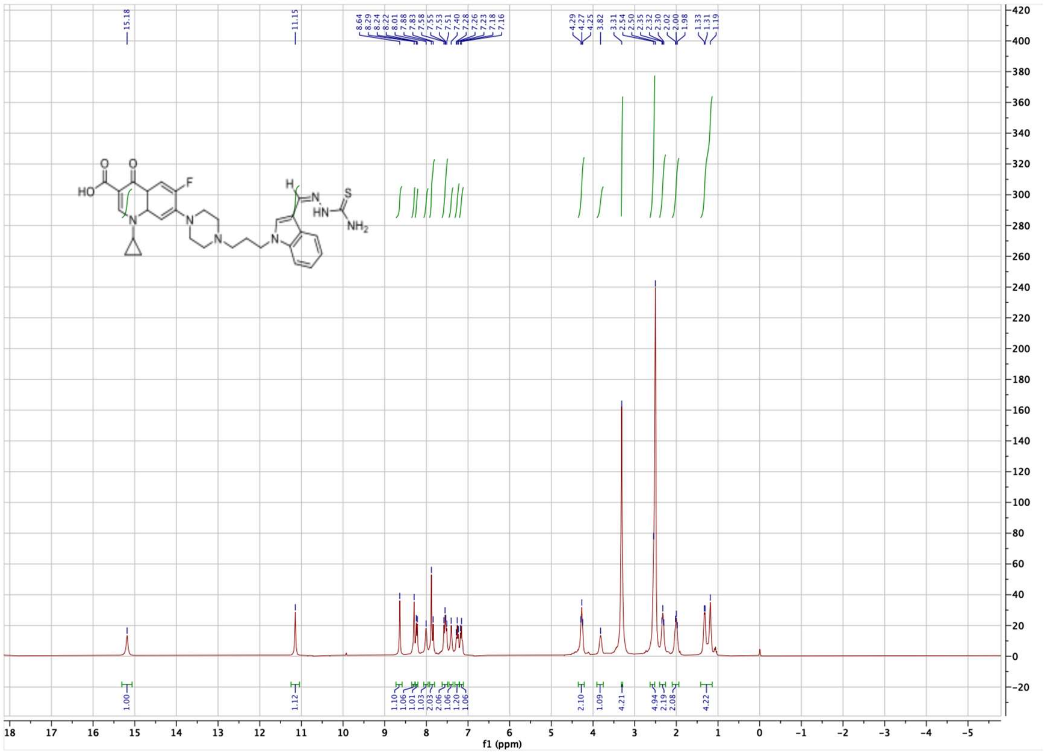

^{1}H NMR of compound ***3b***

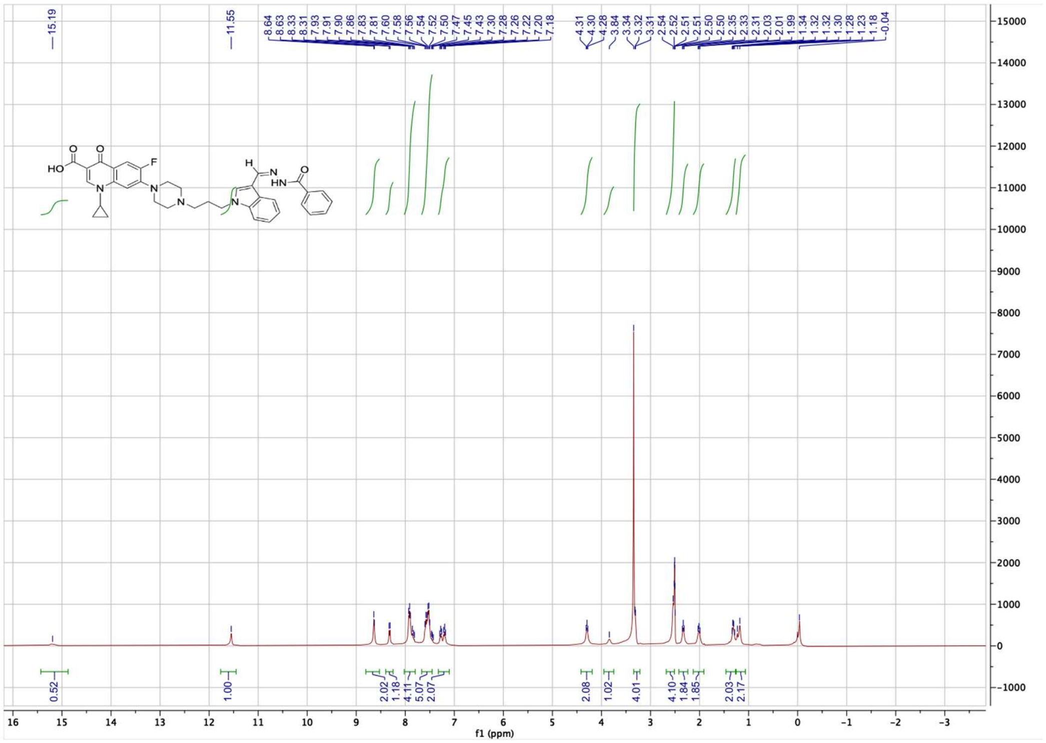

*^{1}H NMR of compound **3c***

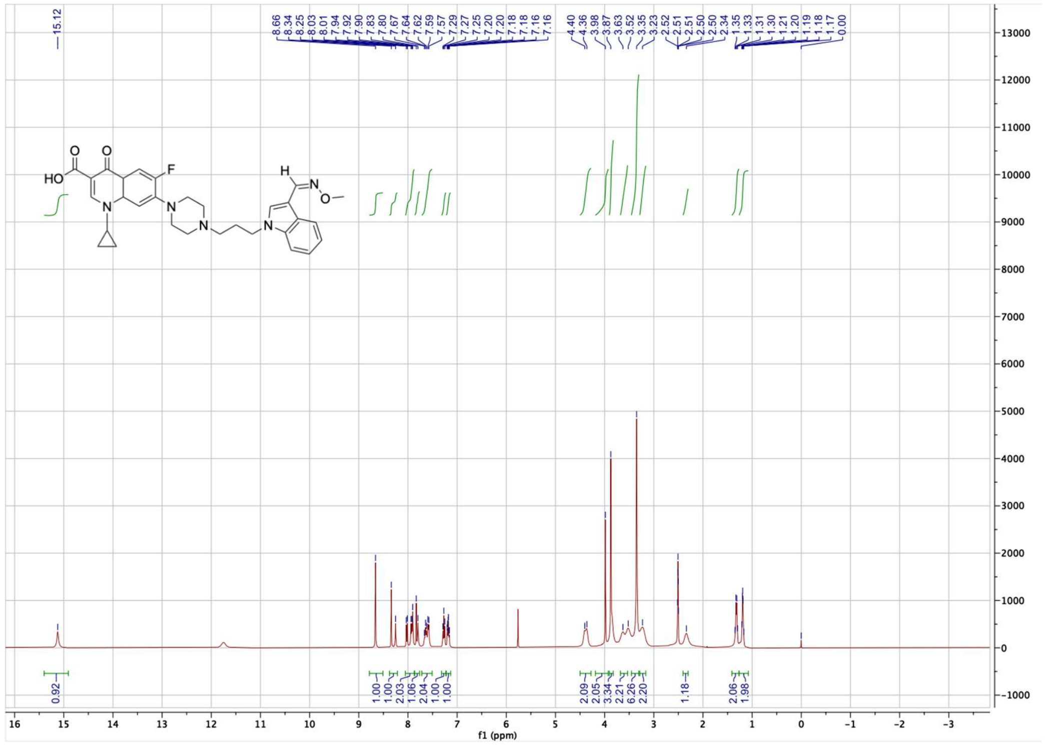

*^{1}H NMR of compound **3d***

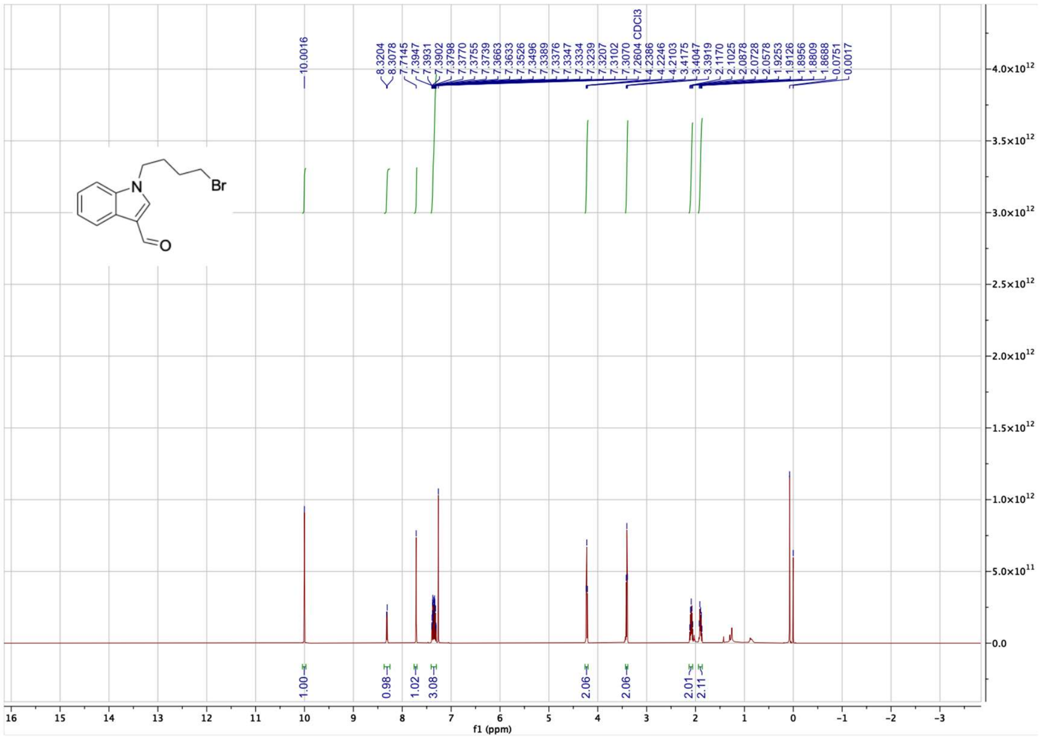

*^{1}H NMR of compound **4a***

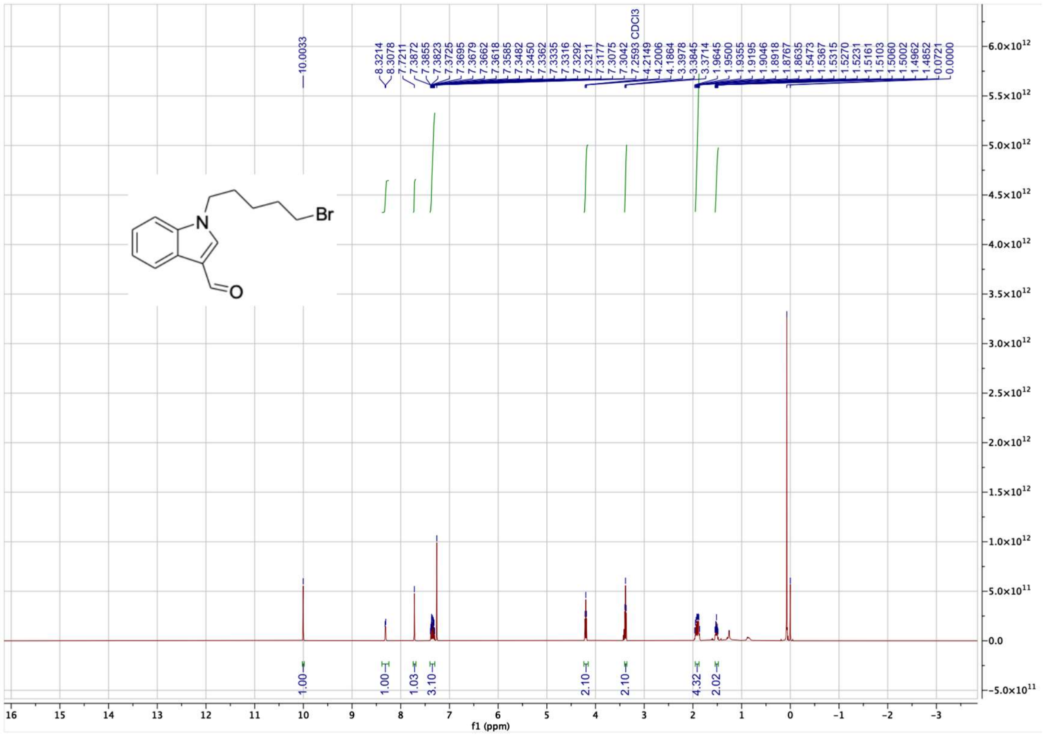

*^{1}H NMR of compound **4b***

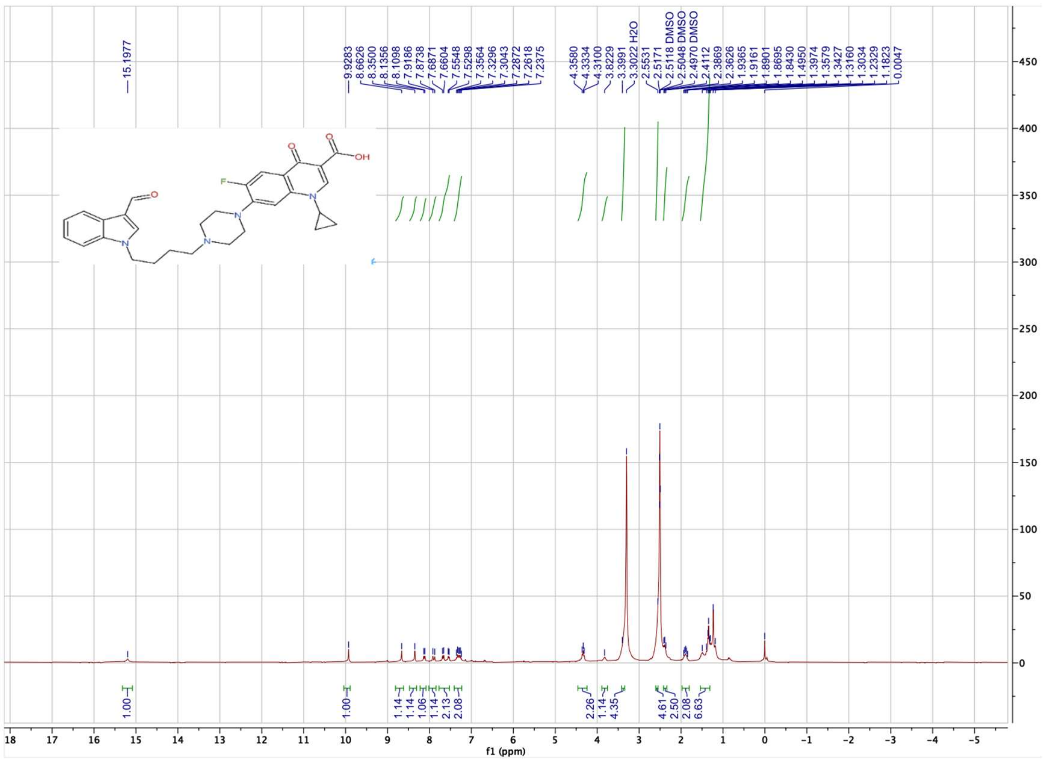

^{1}H NMR of compound ***5a***

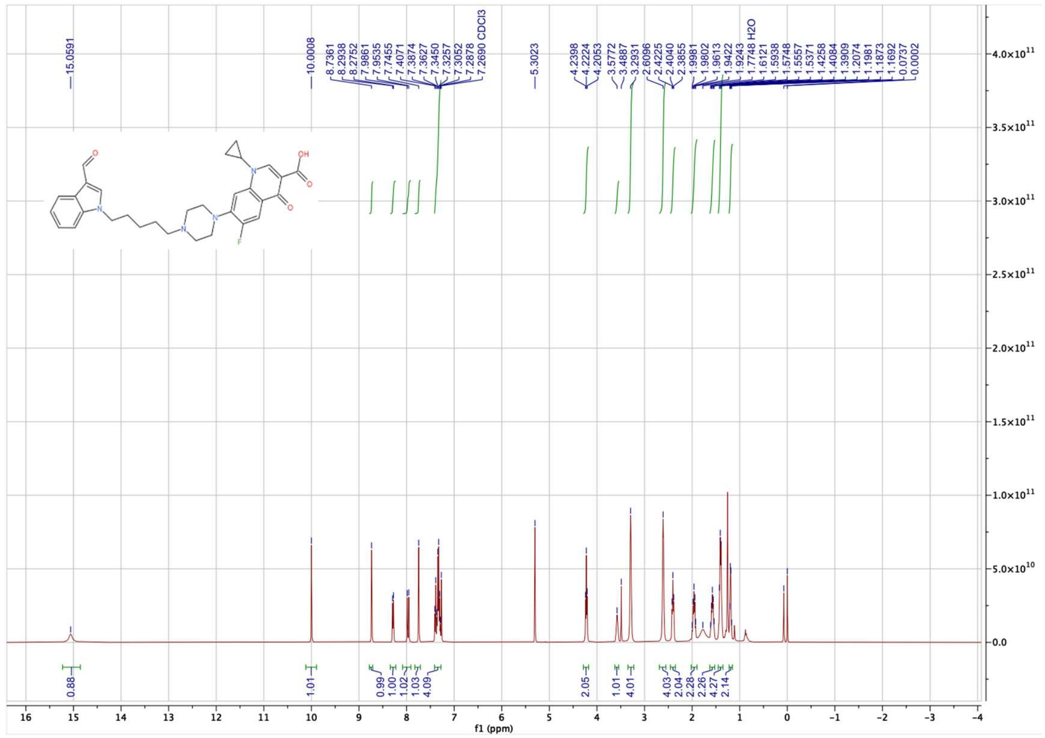

^{1}H NMR of compound ***5b***

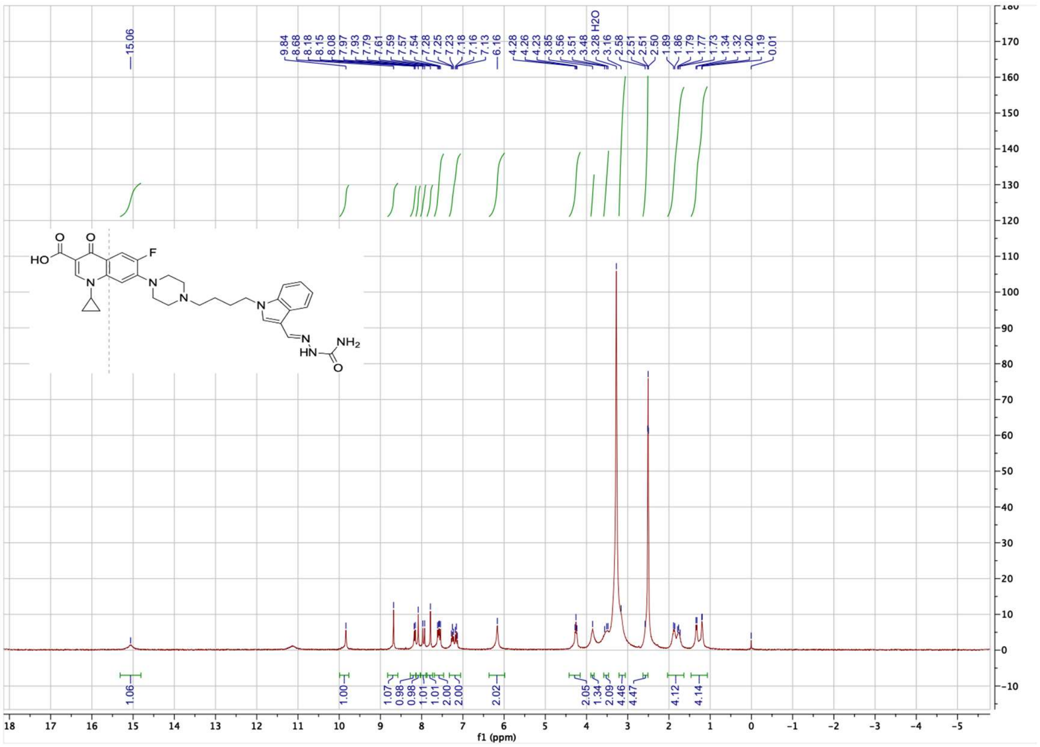

*^{1}H NMR of compound **6a***

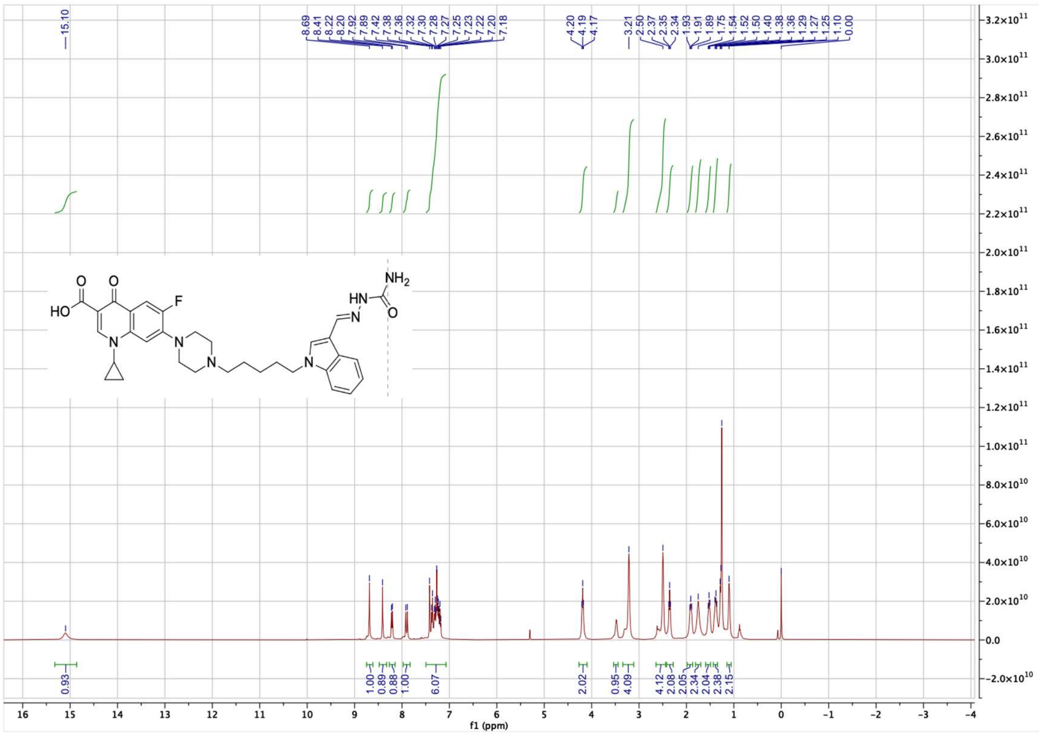

*^{1}H NMR of compound **6b***

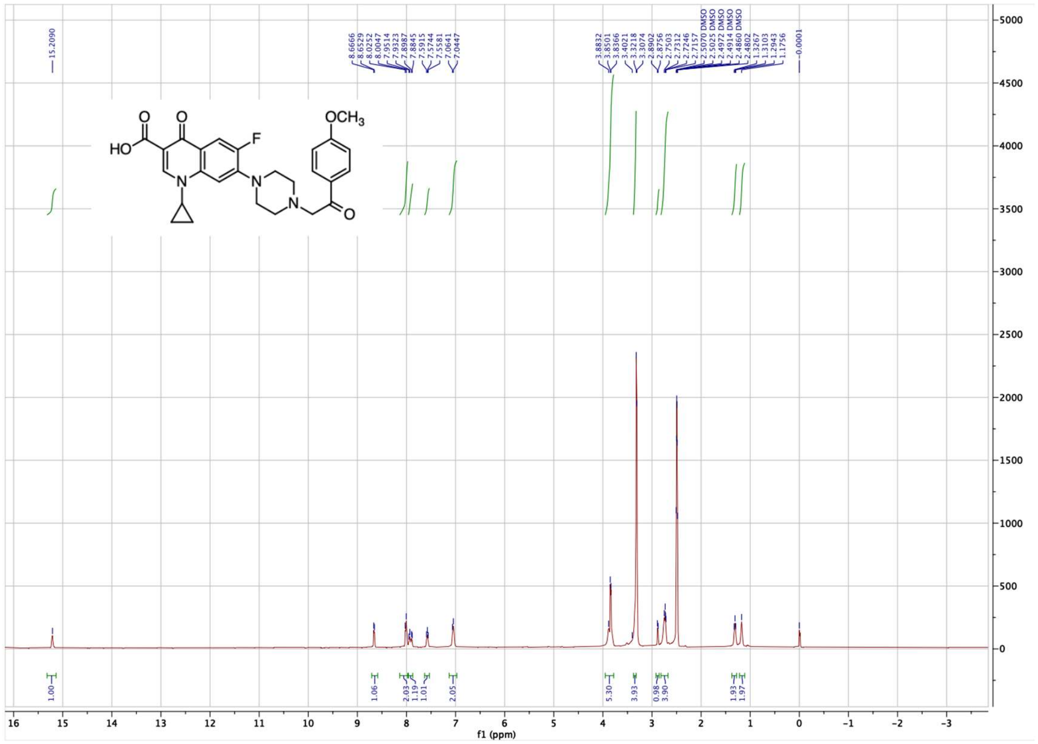

^1H NMR of compound **7a**

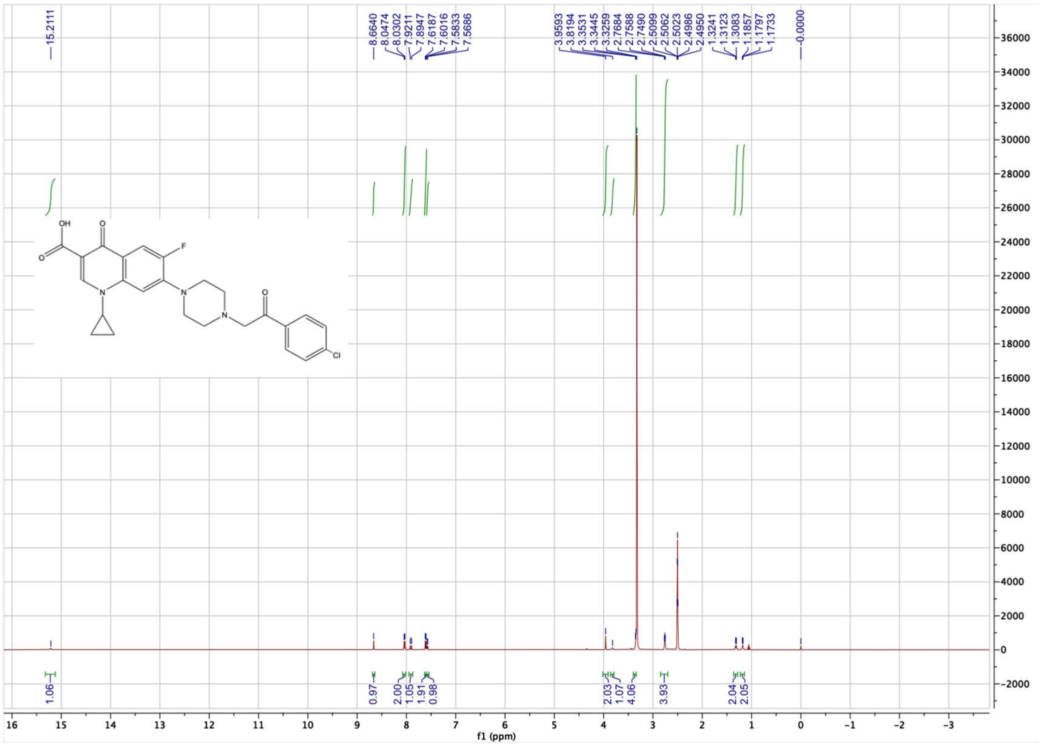

^1H NMR of compound **7b**

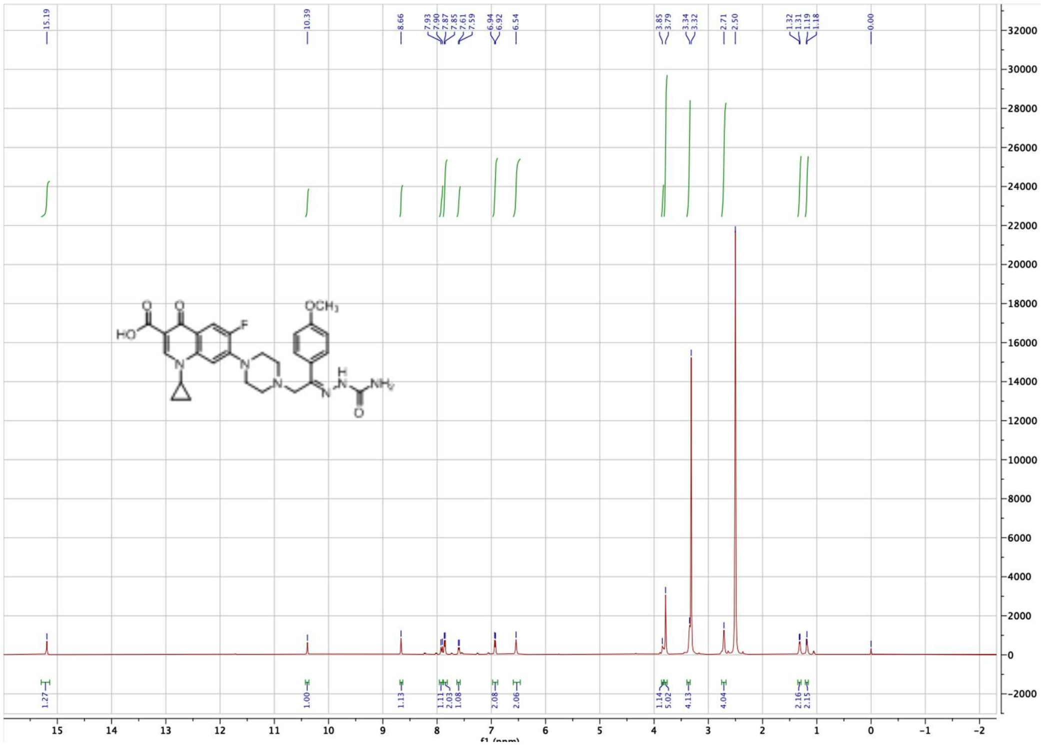

*^{1}H NMR of compound **8a***

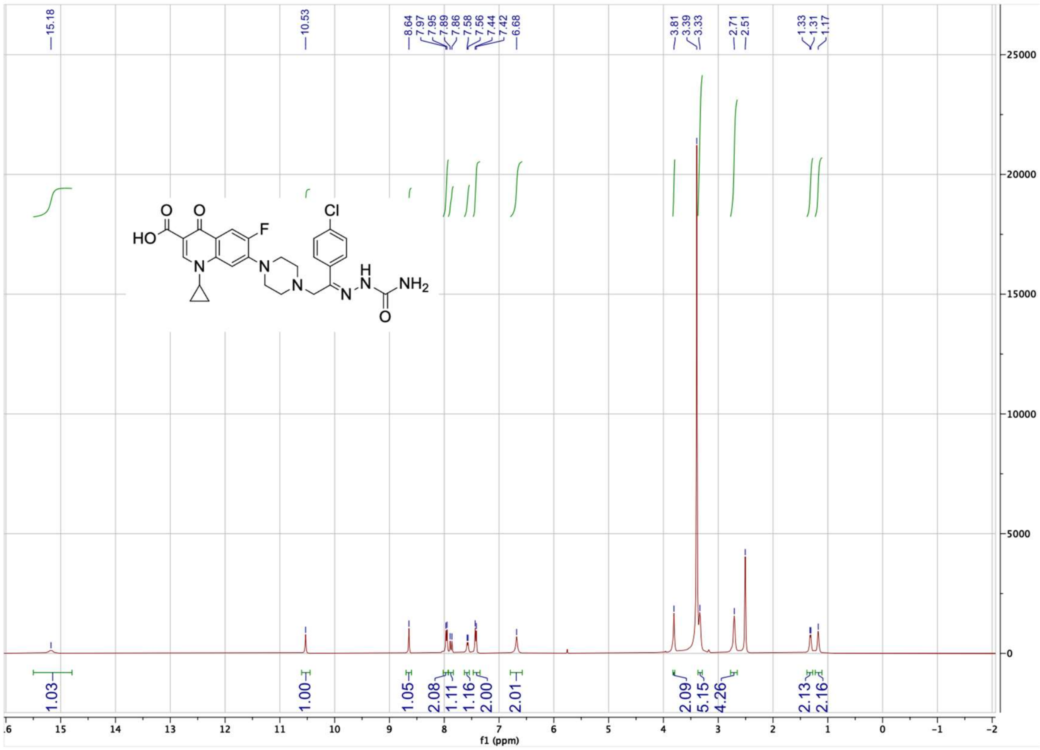

*^{1}H NMR of compound **8b***

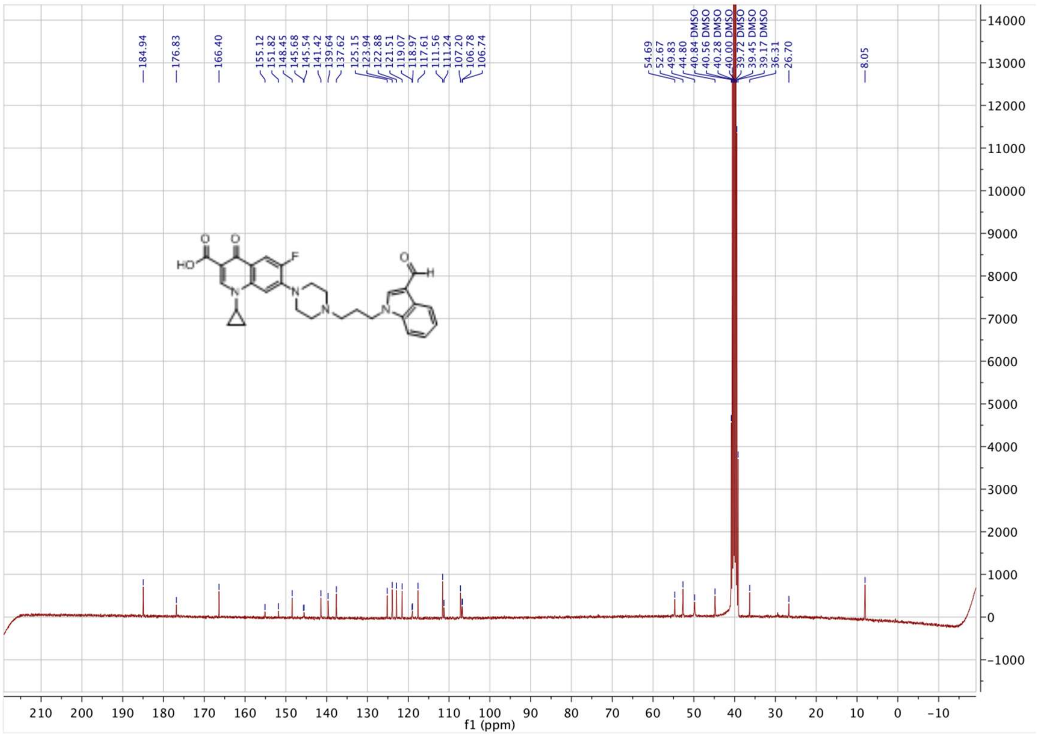

^{13}C NMR of compound **2**

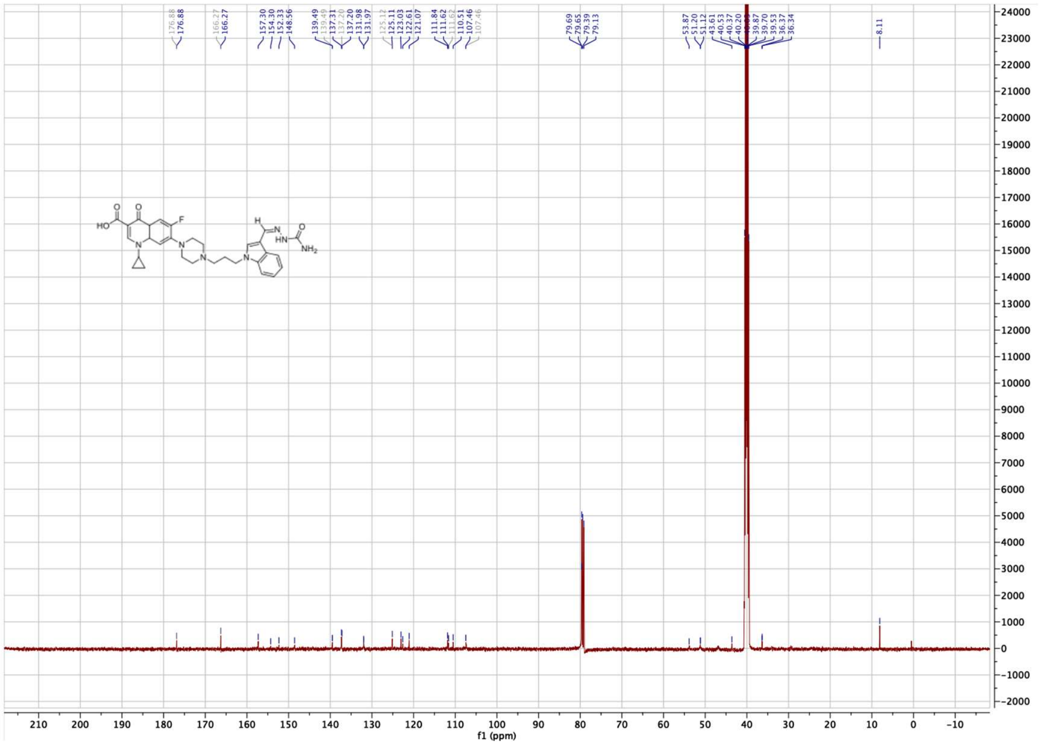

^{13}C NMR of compound **3a**

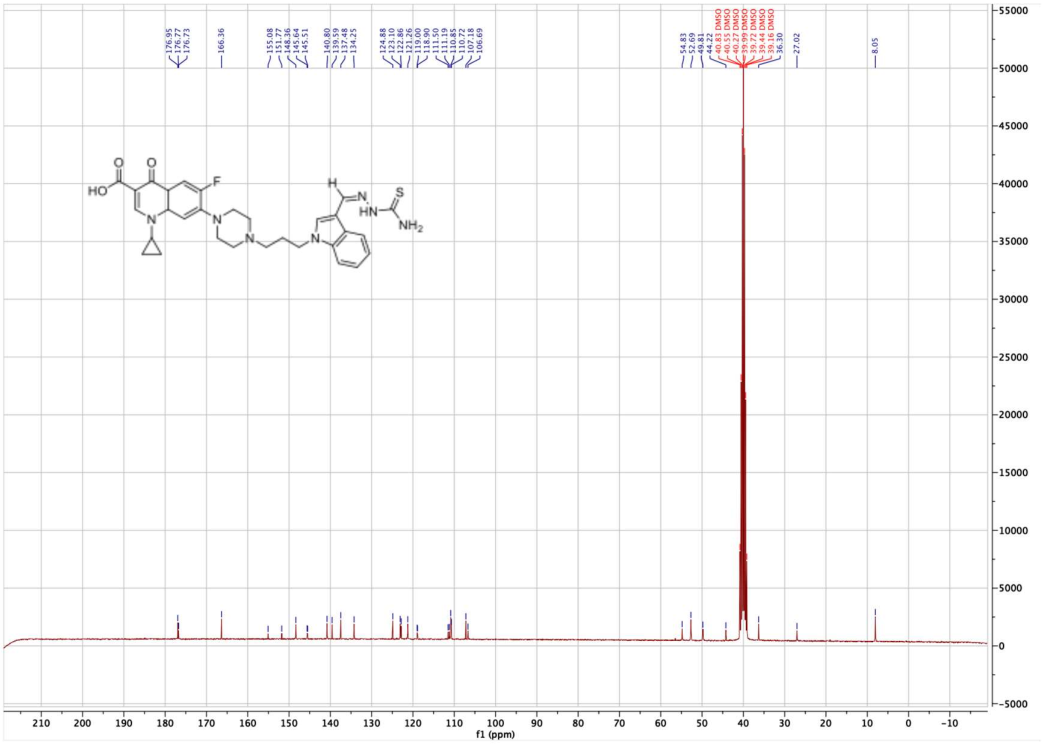

^{13}C NMR of compound ***3b***

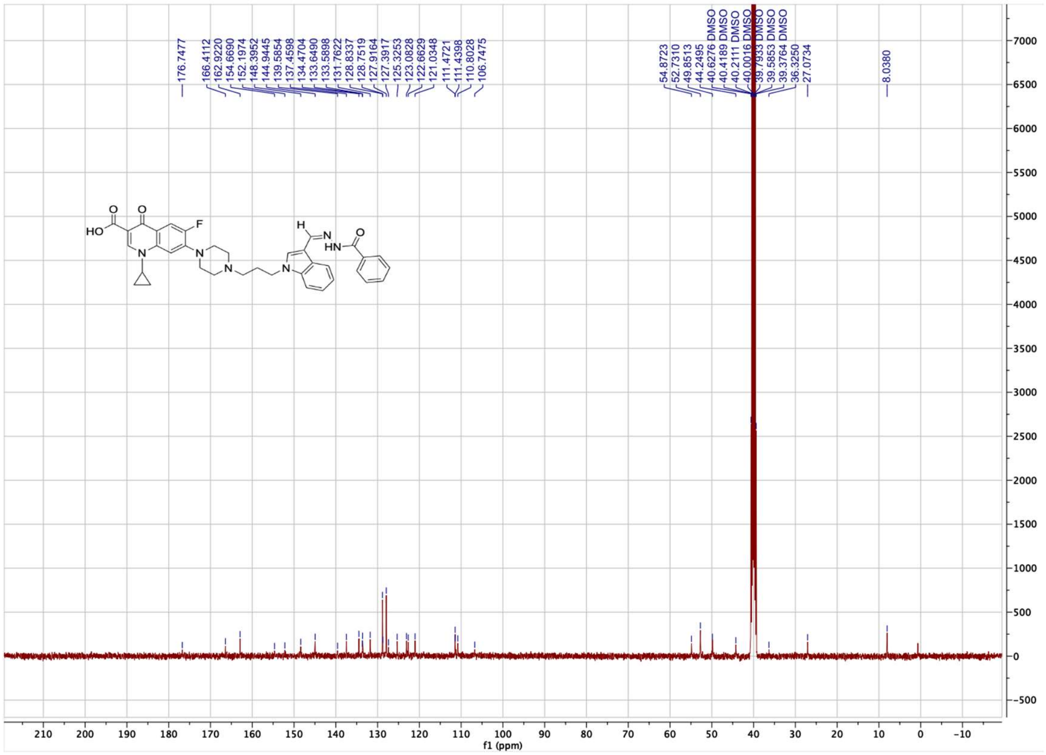

^{13}C NMR of compound ***3c***

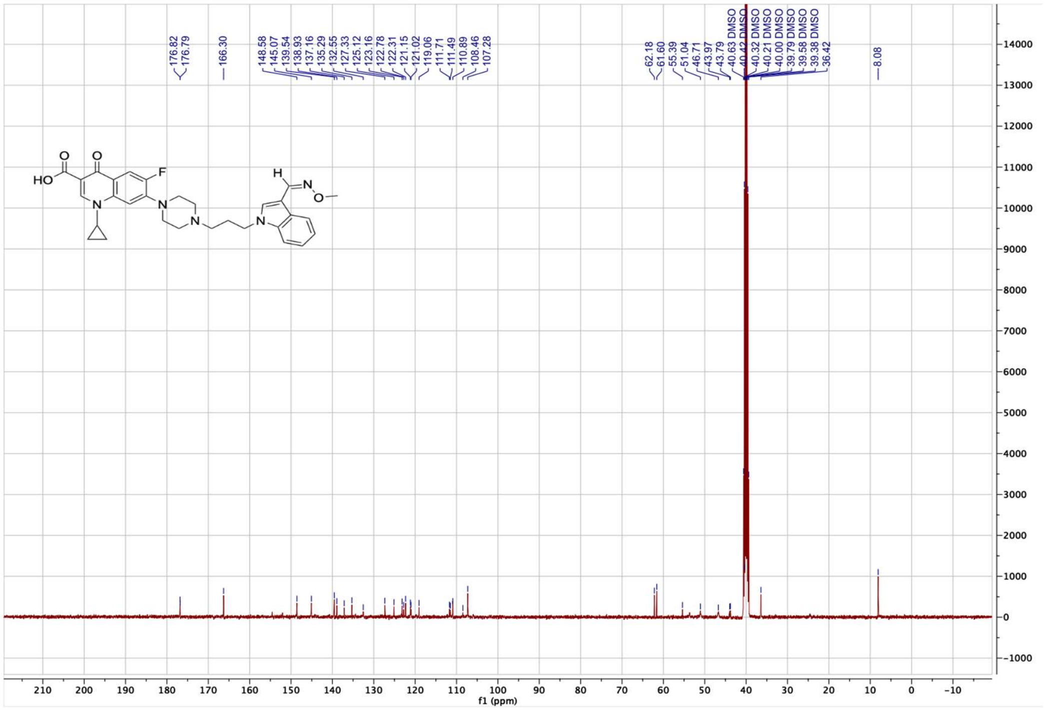

*^{13}C NMR of compound **3d***

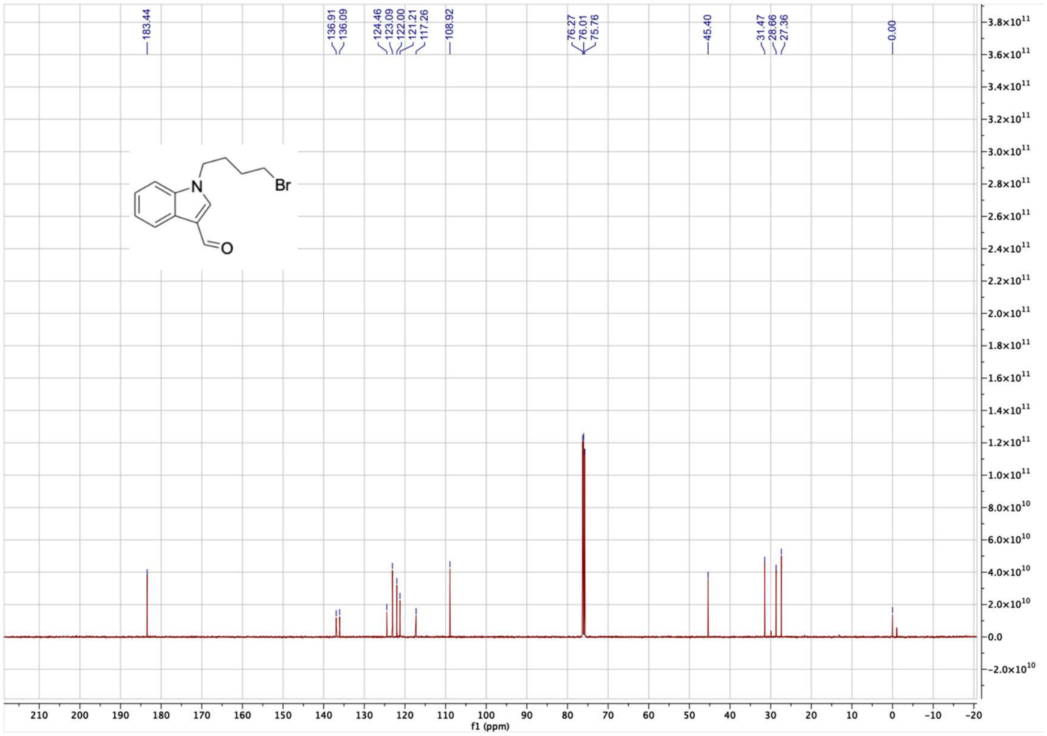

*^{13}C NMR of compound **4a***

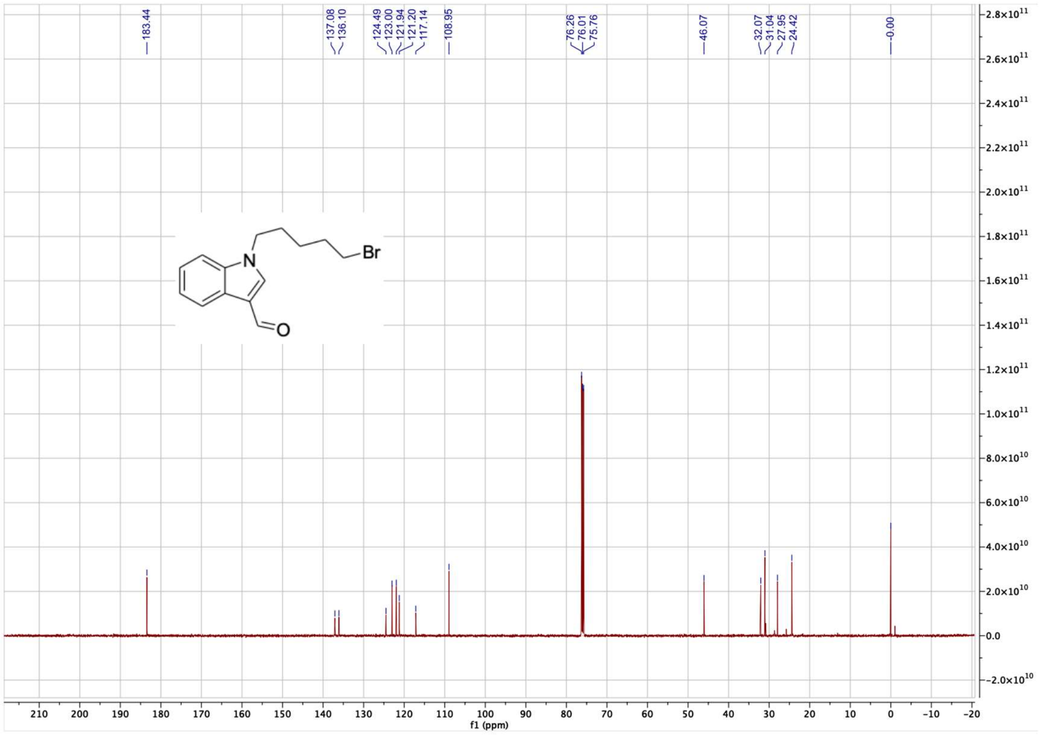

*^{13}C NMR of compound **4b***

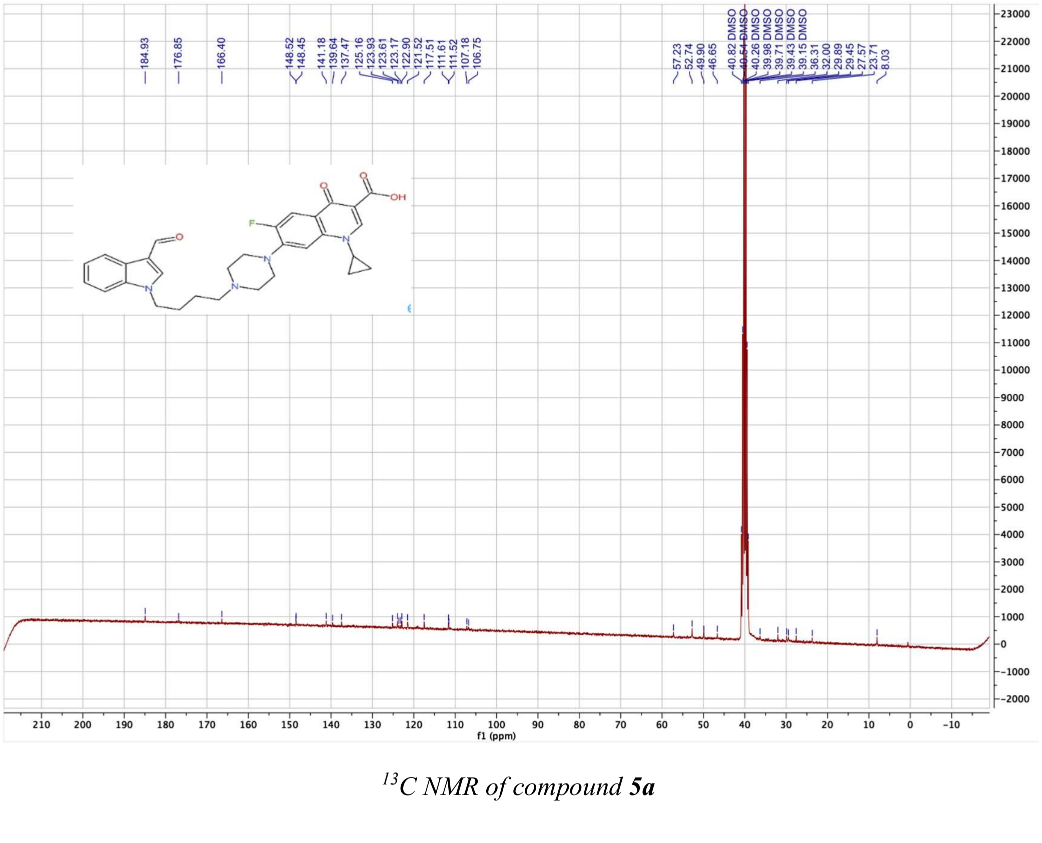

*^{13}C NMR of compound **5a***

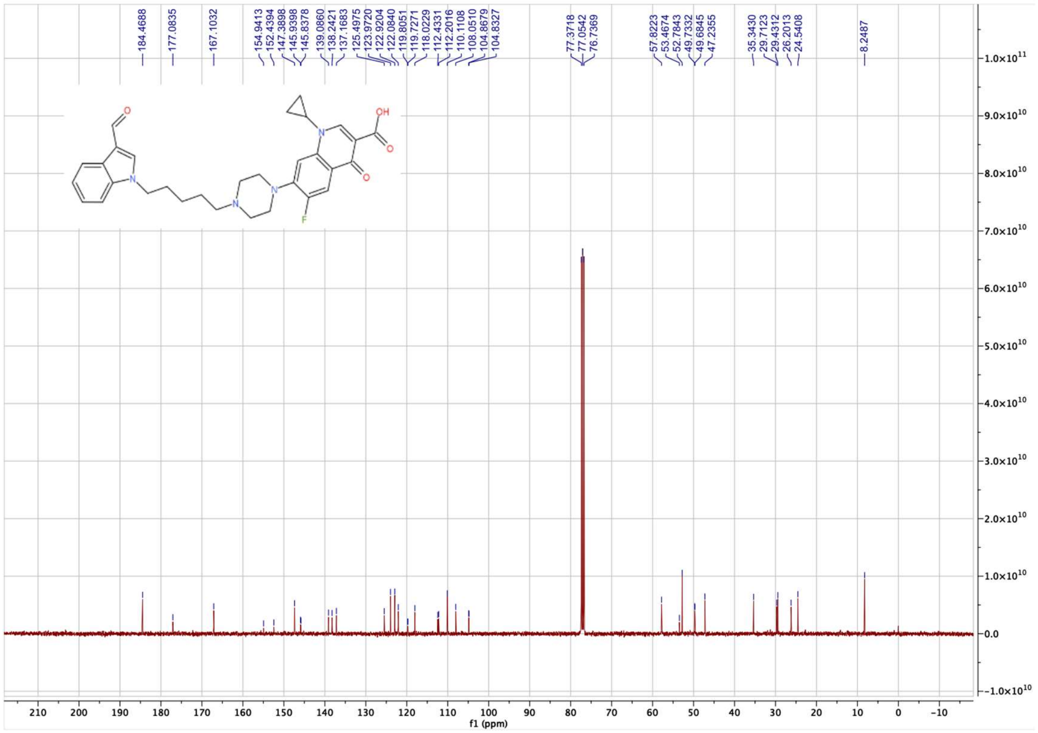

^{13}C NMR of compound ***5b***

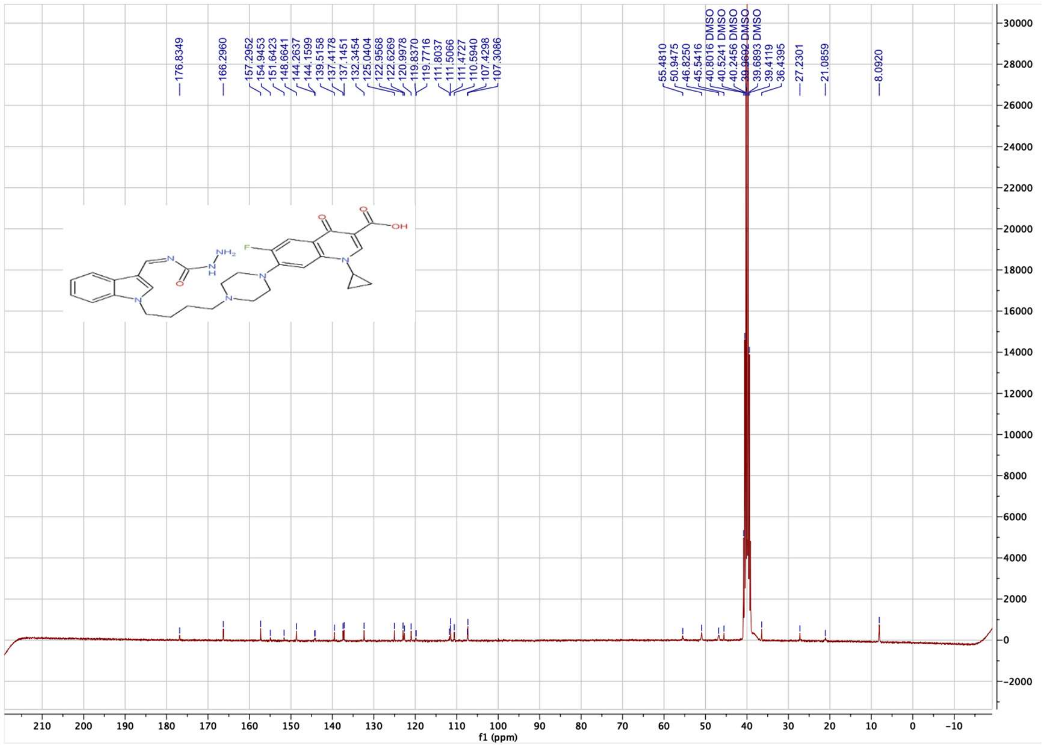

^{13}C NMR of compound ***6a***

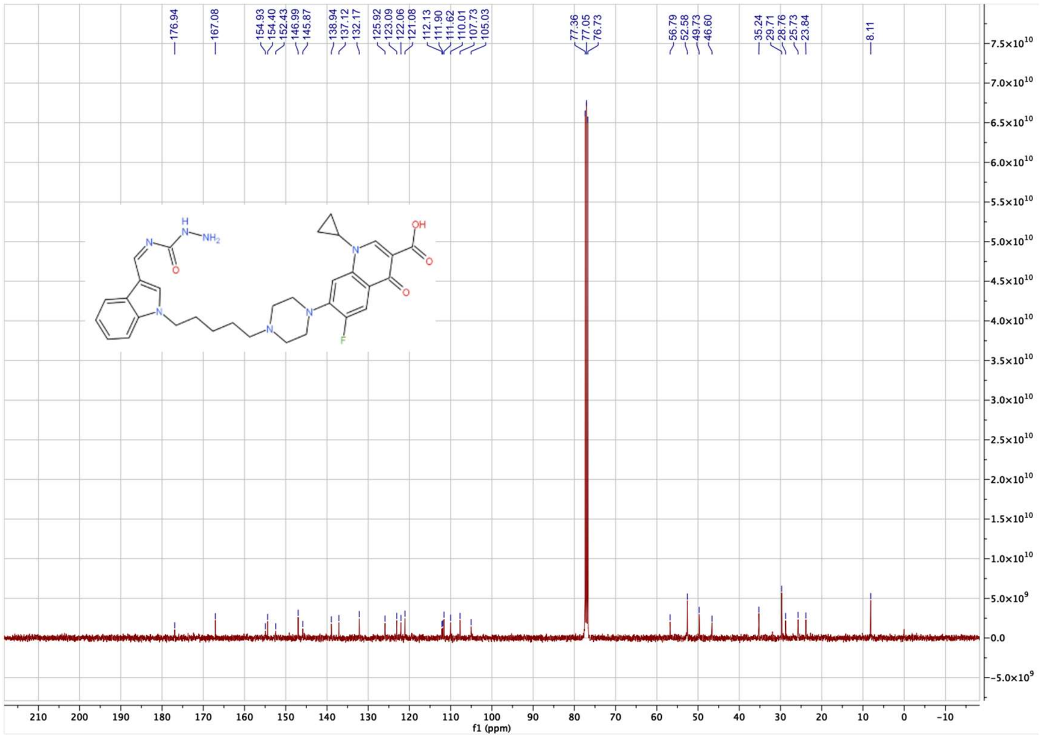

*^{13}C NMR of compound **6b***

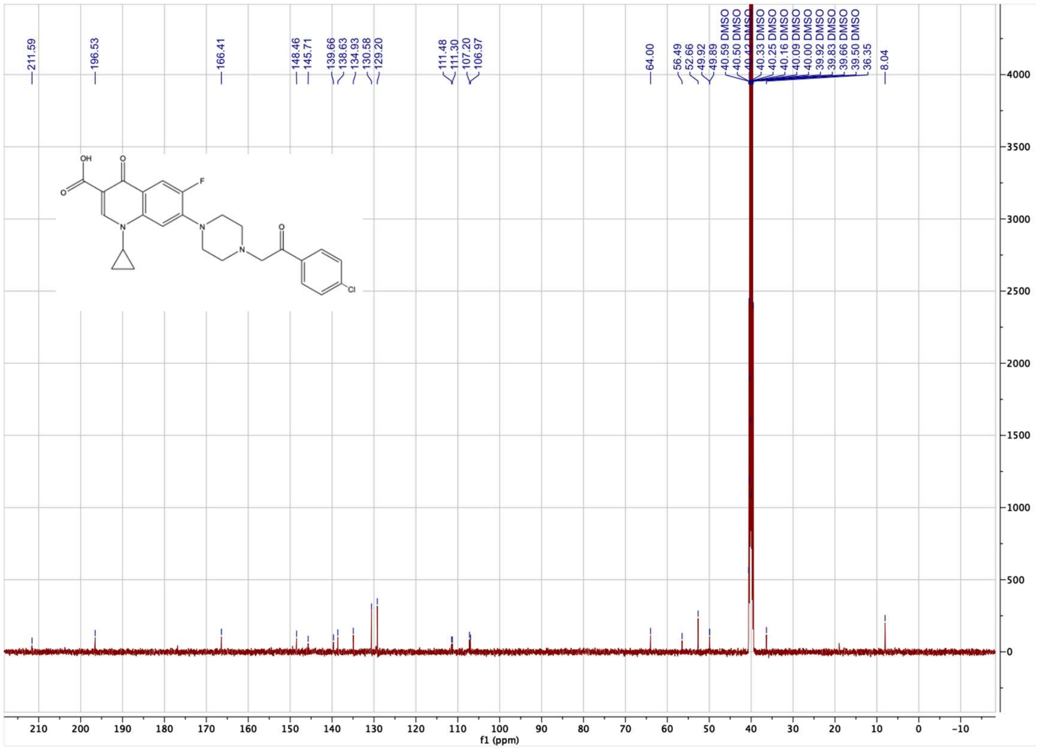

*^{13}C NMR of compound **7b***

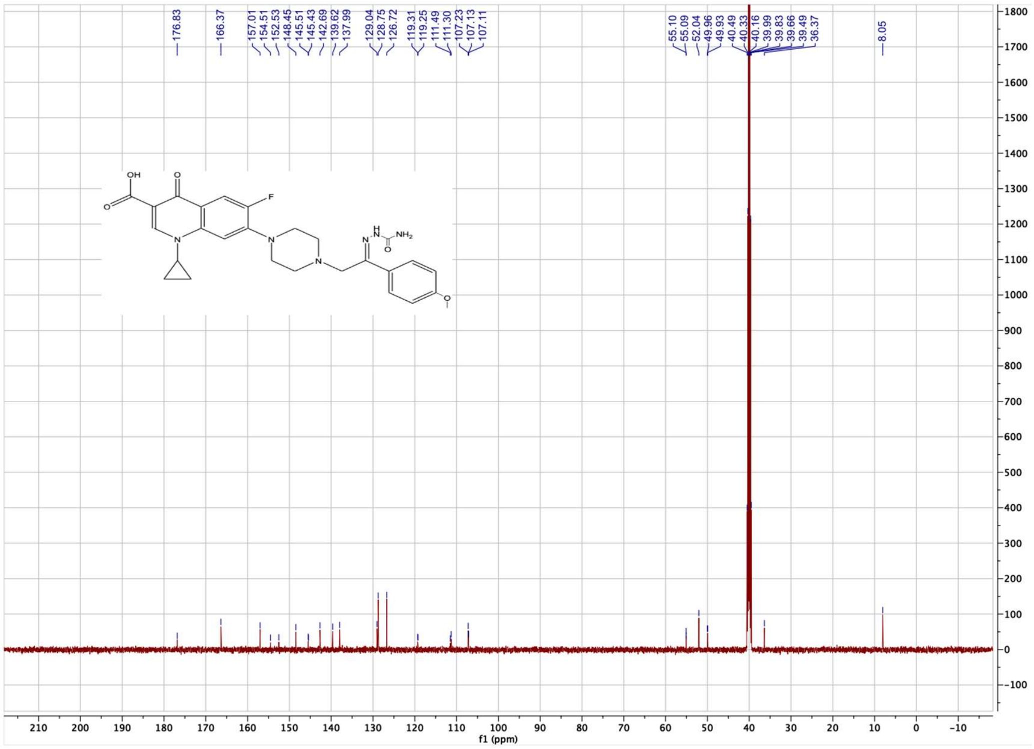

^{13}C NMR of compound ***8a***

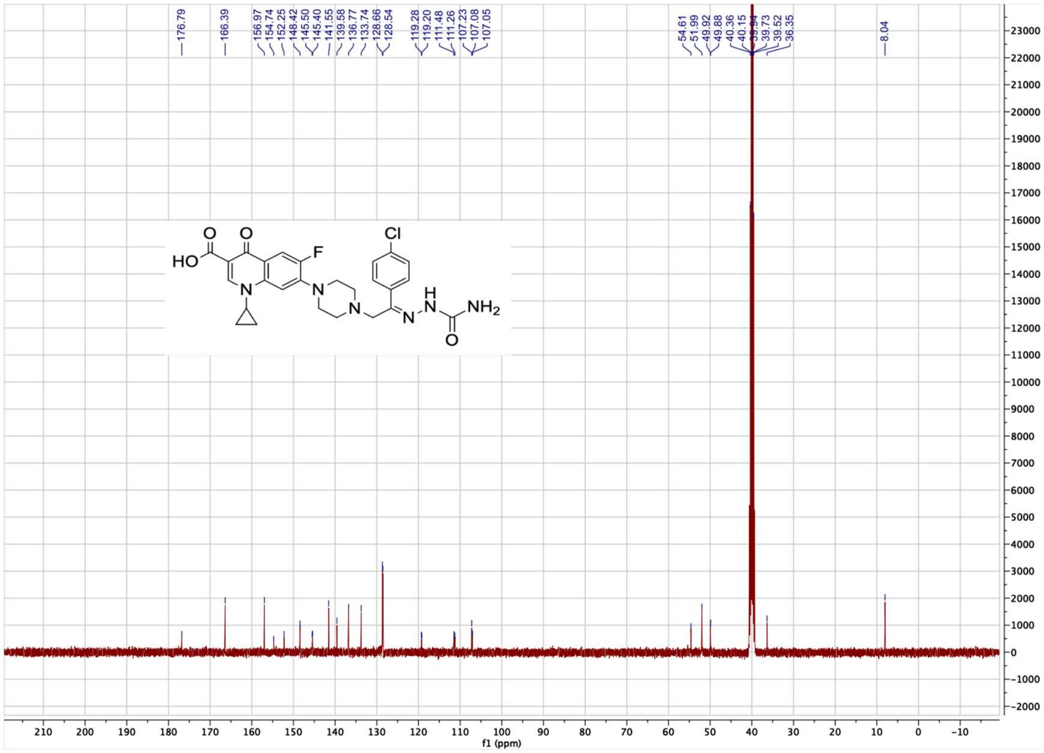

^{13}C NMR of compound ***8b***

Elemental Composition Report **Page 1**

Single Mass Analysis
Tolerance = 20.0 PPM / DBE: min = -1.5, max = 50.0
Element prediction: Off
Number of isotope peaks used for i-FIT = 3

Monoisotopic Mass, Even Electron Ions
30 formula(e) evaluated with 1 results within limits (up to 50 closest results for each mass)
Elements Used:
C: 29-29 H: 10-35 N: 0-5 O: 0-7 F: 1-1

e
0502-4-M-2 24 (0.151) 1: TOF MS ES+
8.71e+006

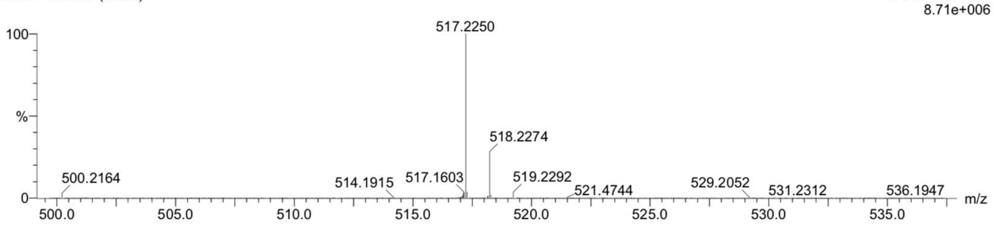

Minimum:				-1.5				
Maximum:		5.0	20.0	50.0				

Mass	Calc. Mass	mDa	PPM	DBE	i-FIT	Norm	Conf(%)	Formula
517.2250	517.2251	-0.1	-0.2	16.5	1035.2	n/a	n/a	C29 H30 N4 O4 F

*HR-MS of compound **2***

Elemental Composition Report **Page 1**

Single Mass Analysis
Tolerance = 20.0 PPM / DBE: min = -1.5, max = 50.0
Element prediction: Off
Number of isotope peaks used for i-FIT = 3

Monoisotopic Mass, Even Electron Ions
50 formula(e) evaluated with 1 results within limits (up to 50 closest results for each mass)
Elements Used:
C: 29-30 H: 10-35 N: 0-7 O: 0-7 F: 1-1
e
0502-4-M-3A 46 (0.274) 1: TOF MS ES+
1.65e+005

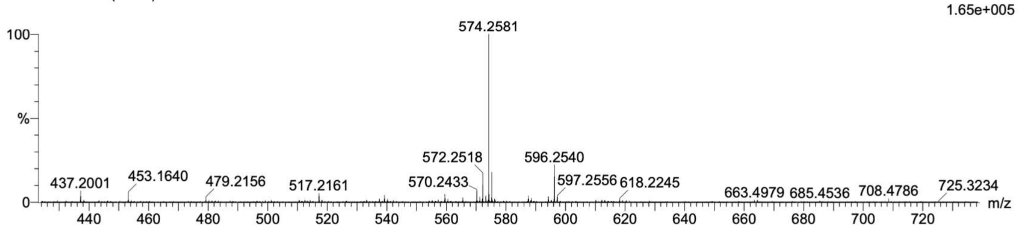

Minimum:				-1.5				
Maximum:		5.0	20.0	50.0				

Mass	Calc. Mass	mDa	PPM	DBE	i-FIT	Norm	Conf(%)	Formula
574.2581	574.2578	0.3	0.5	17.5	817.6	n/a	n/a	C30 H33 N7 O4 F

*HR-MS of compound **3a***

Elemental Composition Report Page 1

Single Mass Analysis
Tolerance = 20.0 PPM / DBE: min = -1.5, max = 50.0
Element prediction: Off
Number of isotope peaks used for i-FIT = 3

Monoisotopic Mass, Even Electron Ions
46 formula(e) evaluated with 1 results within limits (up to 50 closest results for each mass)
Elements Used:
C: 29-30 H: 10-35 N: 0-7 O: 0-7 F: 1-1 S: 1-1
e
0502-4-M-3B 24 (0.151) 1: TOF MS ES+ 1.89e+006

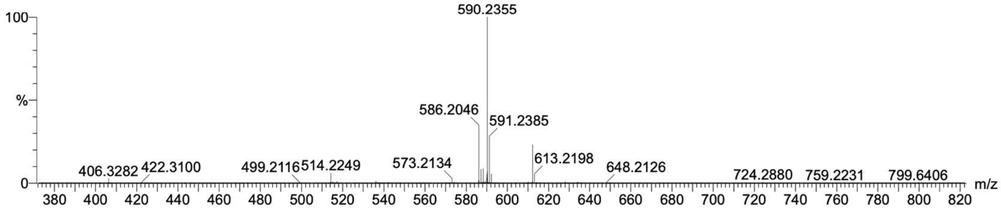

Minimum:				-1.5				
Maximum:		5.0	20.0	50.0				
Mass	Calc. Mass	mDa	PPM	DBE	i-FIT	Norm	Conf(%)	Formula
590.2355	590.2350	0.5	0.8	17.5	822.5	n/a	n/a	C30 H33 N7 O3 F S

*HR-MS of compound **3b***

Elemental Composition Report Page 1

Single Mass Analysis
Tolerance = 20.0 PPM / DBE: min = -1.5, max = 50.0
Element prediction: Off
Number of isotope peaks used for i-FIT = 3

Monoisotopic Mass, Even Electron Ions
26 formula(e) evaluated with 1 results within limits (up to 50 closest results for each mass)
Elements Used:
C: 36-36 H: 30-40 N: 0-7 O: 0-7 F: 1-1
e
0502-4-M-3C 39 (0.238) 1: TOF MS ES+ 1.70e+005

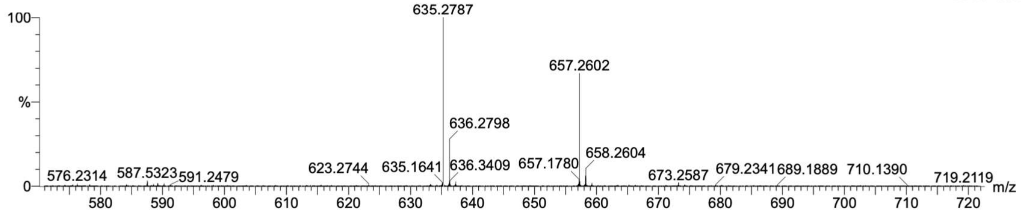

Minimum:				-1.5				
Maximum:		5.0	20.0	50.0				
Mass	Calc. Mass	mDa	PPM	DBE	i-FIT	Norm	Conf(%)	Formula
635.2787	635.2782	0.5	0.8	21.5	703.7	n/a	n/a	C36 H36 N6 O4 F

*HR-MS of compound **3c***

Elemental Composition Report **Page 1**

Single Mass Analysis
Tolerance = 20.0 PPM / DBE: min = -1.5, max = 50.0
Element prediction: Off
Number of isotope peaks used for i-FIT = 3

Monoisotopic Mass, Even Electron Ions
25 formula(e) evaluated with 1 results within limits (up to 50 closest results for each mass)
Elements Used:
C: 30-30 H: 30-40 N: 0-7 O: 0-7 F: 1-1
e
0502-4-M-3D 21 (0.135) 1: TOF MS ES+ 4.19e+006

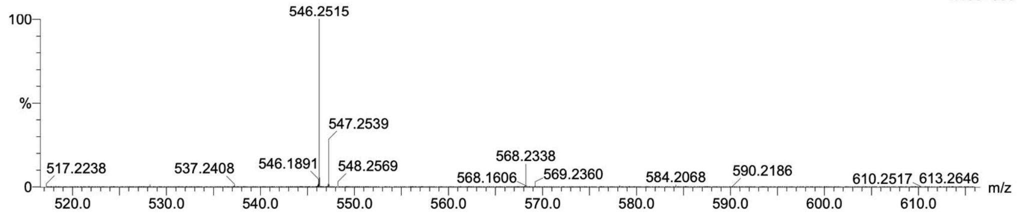

Minimum:				-1.5				
Maximum:		5.0	20.0	50.0				

Mass	Calc. Mass	mDa	PPM	DBE	i-FIT	Norm	Conf(%)	Formula
546.2515	546.2517	-0.2	-0.4	16.5	958.2	n/a	n/a	C30 H33 N5 O4 F

HR-MS of compound ***3d***

Elemental Composition Report **Page 1**

Single Mass Analysis
Tolerance = 20.0 PPM / DBE: min = -1.5, max = 50.0
Element prediction: Off
Number of isotope peaks used for i-FIT = 3

Monoisotopic Mass, Even Electron Ions
26 formula(e) evaluated with 1 results within limits (up to 50 closest results for each mass)
Elements Used:
C: 30-30 H: 30-40 N: 0-7 O: 0-7 F: 1-1
e
0502-4-M-5A 23 (0.146) 1: TOF MS ES+ 4.67e+006

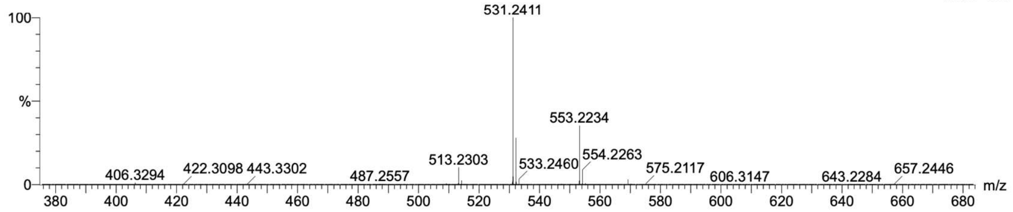

Minimum:				-1.5				
Maximum:		5.0	20.0	50.0				

Mass	Calc. Mass	mDa	PPM	DBE	i-FIT	Norm	Conf(%)	Formula
531.2411	531.2408	0.3	0.6	16.5	941.8	n/a	n/a	C30 H32 N4 O4 F

HR-MS of compound ***5a***

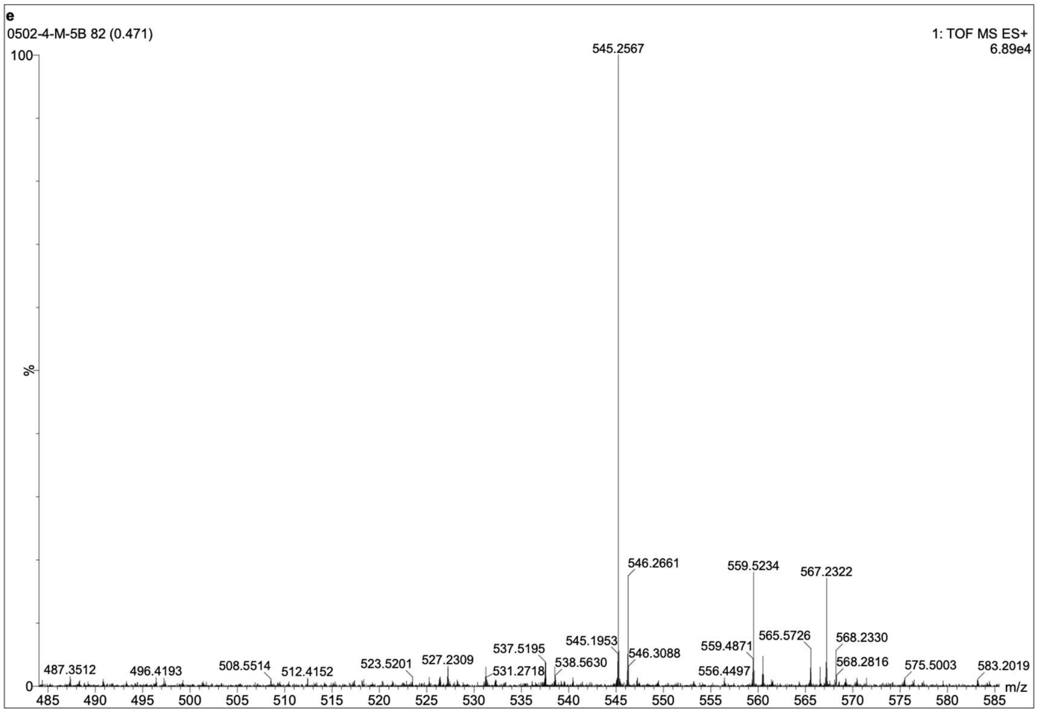

HR-MS of compound ***5b***

Elemental Composition Report **Page 1**

Single Mass Analysis
Tolerance = 20.0 PPM / DBE: min = -1.5, max = 50.0
Element prediction: Off
Number of isotope peaks used for i-FIT = 3

Monoisotopic Mass, Even Electron Ions
3 formula(e) evaluated with 1 results within limits (up to 50 closest results for each mass)
Elements Used:
C: 31-32 H: 30-40 N: 7-7 O: 0-7 F: 1-1

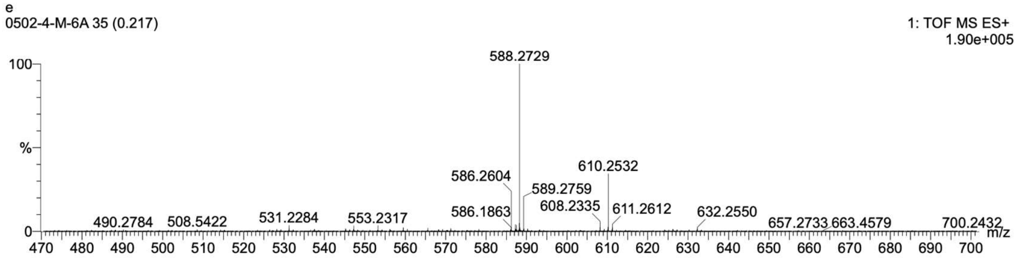

Minimum:				-1.5				
Maximum:		5.0	20.0	50.0				
Mass	Calc. Mass	mDa	PPM	DBE	i-FIT	Norm	Conf(%)	Formula
588.2729	588.2735	-0.6	-1.0	17.5	701.8	n/a	n/a	C31 H35 N7 O4 F

HR-MS of compound ***6a***

Elemental Composition Report **Page 1**

Single Mass Analysis
Tolerance = 20.0 PPM / DBE: min = -1.5, max = 50.0
Element prediction: Off
Number of isotope peaks used for i-FIT = 3

Monoisotopic Mass, Even Electron Ions
2 formula(e) evaluated with 1 results within limits (up to 50 closest results for each mass)
Elements Used:
C: 31-32 H: 30-40 N: 7-7 O: 0-7 F: 1-1

e
0502-4-M-6B 81 (0.466) 1: TOF MS ES+
2.69e+002

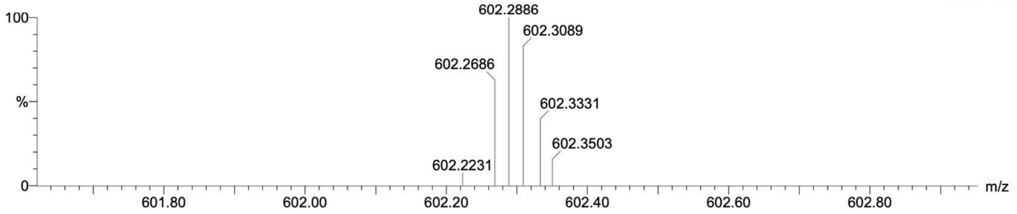

Minimum:				-1.5				
Maximum:		5.0	20.0	50.0				

Mass	Calc. Mass	mDa	PPM	DBE	i-FIT	Norm	Conf(%)	Formula
602.2886	602.2891	-0.5	-0.8	17.5	49.0	n/a	n/a	C32 H37 N7 O4 F

*HR-MS of compound **6b***

Elemental Composition Report **Page 1**

Single Mass Analysis
Tolerance = 20.0 PPM / DBE: min = -1.5, max = 50.0
Element prediction: Off
Number of isotope peaks used for i-FIT = 3

Monoisotopic Mass, Even Electron Ions
43 formula(e) evaluated with 1 results within limits (up to 50 closest results for each mass)
Elements Used:
C: 26-30 H: 27-40 N: 0-7 O: 0-7 F: 1-1

2
0502-4-M-10A 29 (0.177) 1: TOF MS ES+
3.43e+006

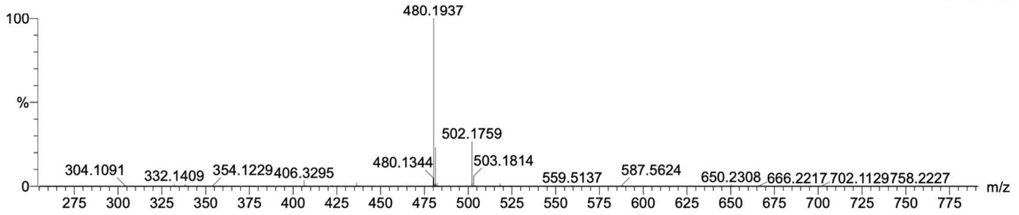

Minimum:				-1.5				
Maximum:		5.0	20.0	50.0				

Mass	Calc. Mass	mDa	PPM	DBE	i-FIT	Norm	Conf(%)	Formula
480.1937	480.1935	0.2	0.4	14.5	1091.4	n/a	n/a	C26 H27 N3 O5 F

*HR-MS of compound **7a***

Elemental Composition Report

Single Mass Analysis
Tolerance = 20.0 PPM / DBE: min = -1.5, max = 50.0
Element prediction: Off
Number of isotope peaks used for i-FIT = 3

Monoisotopic Mass, Even Electron Ions
52 formula(e) evaluated with 1 results within limits (up to 50 closest results for each mass)
Elements Used:
C: 25-29 H: 24-40 N: 0-7 O: 0-7 F: 1-1 Cl: 1-1

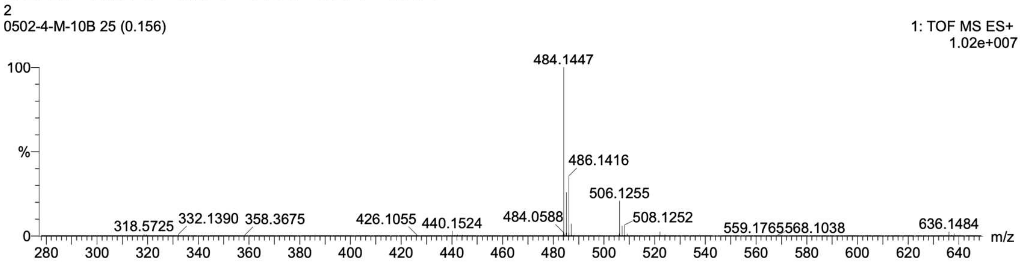

Minimum:				-1.5				
Maximum:		5.0	20.0	50.0				
Mass	Calc. Mass	mDa	PPM	DBE	i-FIT	Norm	Conf(%)	Formula
484.1447	484.1439	0.8	1.7	14.5	1214.7	n/a	n/a	C25 H24 N3 O4 F Cl

*HR-MS of compound **7b***

Elemental Composition Report

Single Mass Analysis
Tolerance = 20.0 PPM / DBE: min = -1.5, max = 50.0
Element prediction: Off
Number of isotope peaks used for i-FIT = 3

Monoisotopic Mass, Even Electron Ions
40 formula(e) evaluated with 1 results within limits (up to 50 closest results for each mass)
Elements Used:
C: 27-27 H: 24-40 N: 0-7 O: 0-7 F: 1-1

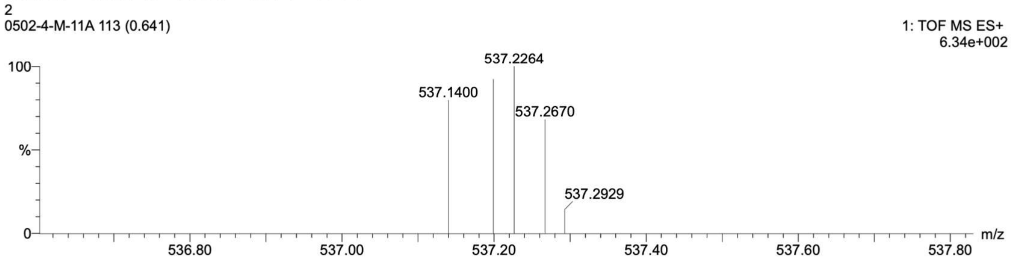

Minimum:				-1.5				
Maximum:		5.0	20.0	50.0				
Mass	Calc. Mass	mDa	PPM	DBE	i-FIT	Norm	Conf(%)	Formula
537.2264	537.2262	0.2	0.4	15.5	44.4	n/a	n/a	C27 H30 N6 O5 F

*HR-MS of compound **8a***

Elemental Composition Report

Single Mass Analysis

Tolerance = 20.0 PPM / DBE: min = -1.5, max = 50.0
Element prediction: Off
Number of isotope peaks used for i-FIT = 3

Monoisotopic Mass, Even Electron Ions
11 formula(e) evaluated with 1 results within limits (up to 50 closest results for each mass)
Elements Used:
C: 26-27 H: 24-40 N: 5-7 O: 0-7 F: 1-1 Cl: 1-1

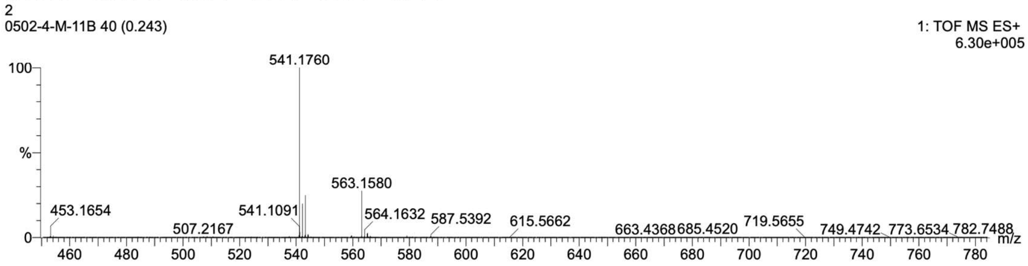

Minimum:				-1.5				
Maximum:		5.0	20.0	50.0				

Mass	Calc. Mass	mDa	PPM	DBE	i-FIT	Norm	Conf(%)	Formula
541.1760	541.1766	-0.6	-1.1	15.5	853.2	n/a	n/a	C26 H27 N6 O4 F Cl

HR-MS of compound ***8b***
